# Monitoring elasmobranch assemblages in a data-poor country from the Eastern Tropical Pacific using baited remote underwater video stations

**DOI:** 10.1038/s41598-020-74282-8

**Published:** 2020-10-14

**Authors:** Mario Espinoza, Tatiana Araya-Arce, Isaac Chaves-Zamora, Isaac Chinchilla, Marta Cambra

**Affiliations:** 1 Centro de Investigación en Ciencias del Mar y Limnología, Universidad de Costa Rica, 2060-11501 San José, Costa Rica; 2 Escuela de Biologia, Universidad de Costa Rica, 2060-11501 San José, Costa Rica; 3 Museo de Zoología, Universidad de Costa Rica, 2060-11501 San José, Costa Rica; 4 Centro de Investigación en Estructuras Microscópicas, Universidad de Costa Rica, 2060-11501 San José, Costa Rica; 5 Área de Conservación Marina Cocos (ACMCO), Sistema Nacional de Áreas de Conservación, Costa Rica, 2060-11501 San José, Costa Rica

**Keywords:** Biodiversity, Conservation biology, Tropical ecology, Zoology, Ichthyology

## Abstract

Understanding how threatened species are distributed in space and time can have direct applications to conservation planning. However, implementing standardized methods to monitor populations of wide-ranging species is often expensive and challenging. In this study, we used baited remote underwater video stations (BRUVS) to quantify elasmobranch abundance and distribution patterns across a gradient of protection in the Pacific waters of Costa Rica. Our BRUVS survey detected 29 species, which represents 54% of the entire elasmobranch diversity reported to date in shallow waters (< 60 m) of the Pacific of Costa Rica. Our data demonstrated that elasmobranchs benefit from no-take MPAs, yet large predators are relatively uncommon or absent from open-fishing sites. We showed that BRUVS are capable of providing fast and reliable estimates of the distribution and abundance of data-poor elasmobranch species over large spatial and temporal scales, and in doing so, they can provide critical information for detecting population-level changes in response to multiple threats such as overfishing, habitat degradation and climate change. Moreover, given that 66% of the species detected are threatened, a well-designed BRUVS survey may provide crucial population data for assessing the conservation status of elasmobranchs. These efforts led to the establishment of a national monitoring program focused on elasmobranchs and key marine megafauna that could guide monitoring efforts at a regional scale.

## Introduction

Developing cost-effective approaches for assessing the population status of large marine predators is crucial given the rapid rate at which some species are declining^1–3^. Both coastal and pelagic fisheries have played a major role in the global decline of elasmobranch species, with an estimated one-quarter of the world’s sharks and rays being currently threatened with extinction^4,5^. Moreover, substantial habitat loss, degradation of critical habitats (e.g. nursery, reproductive and foraging grounds) and climate-driven changes may also impact elasmobranch populations at different spatial and temporal scales^4,6,7^. Therefore, detailed knowledge about elasmobranch distribution patterns and habitat use, particularly how populations of wide-ranging threatened species may be connected^8–11^, remains an essential step to adequately assess their status and trends. Ultimately, understanding how threatened species are distributed in space and time can have direct applications to marine conservation planning^12^. However, developing or implementing standardized methods to monitor populations at multiple scales is often expensive and challenging.

Over the last decade, advances in video technology have led to the production of smaller, more robust, and inexpensive cameras that are becoming a popular among researchers seeking to survey marine life^13^. Remote underwater video survey methods can avoid many of the biases and ecological impacts associated with traditional and extractive sampling methods^14–16^. For example, they can sample over a wide range of habitats that are not suitable for fishing^17,18^, and they are not as restricted by depth and time as most diver-operated methods^15,19^. Recorded footage also provides valuable observations of species’ behaviors in their natural environment^20^, which may have a powerful outreach and educational potential^21^.

The use of baited remote underwater video stations (BRUVS) is perhaps one of the most accessible, highly replicated, non-destructive and effective tool for quantifying fish assemblages, species-habitat associations and anthropogenic impacts across large spatial scales^19,22,23^. Using bait increases the probability of detecting predators in the environment, since the resulting bait plume can trigger bait-search behaviors in nearby species^24,25^. Therefore, this technique has the potential to provide relatively fast baseline data on sharks and rays, an ecologically and economically important group for which basic information on distribution and population trends is often lacking^4,26^.

In some countries of the Eastern Tropical Pacific (ETP) such as Costa Rica, there are major information gaps on elasmobranch population trends, despite a large number of species being threatened and in urgent need of conservation attention^4,27^. Shark landings data from Costa Rica are also scarce and unreliable^28^, and there is limited enforcement of existing management regulations, which has hindered effective conservation actions at national and international levels^27^. Moreover, a long-term study based on diving observations reported significant declines in the abundance of two pelagic sharks (the Scalloped hammerhead shark *Sphyrna lewini* and the silky shark *Carcharhinus falciformis*) in Cocos Island National Park and World Heritage Site of Costa Rica^29^, highlighting that even remote and isolated marine protected areas (MPAs) of the ETP can be susceptible to illegal fishing^29,30^. Therefore, implementing reliable and affordable surveying techniques capable of providing fast baseline data on elasmobranch populations across areas with different levels of protection and enforcement remains crucial to developing sound conservation approaches in the ETP.

Understanding what factors shape elasmobranch diversity and distribution can also have important implications for their management and conservation. The effect of environmental drivers on elasmobranch distribution patterns, abundance and species richness has been widely studied in other regions^31–33^, but only a few studies have been conducted in Costa Rica^34,35^. For example^34^, showed that elasmobranch assemblages from Isla del Coco are negatively associated with the El Niño Southern Oscillation (ENSO) event, whereas^35^ found that depth was one of the main drivers shaping demersal elasmobranch distribution along the continental shelf of the Pacific of Costa Rica. For a few threatened elasmobranchs such as the scalloped hammerhead shark and the largetooth sawfish *Pristis pristis*, there has been recent efforts to understand what factors shape their distribution and abundance patterns^36–38^. However, major information gaps remain for most species, particularly those demersal and reef-associated that are found along the continental shelf.

Establishing long-term monitoring programs capable of mapping the spatial distribution of elasmobranch species over large scales, as well as understanding the role that spatial management and environmental drivers play is crucial to detect population level changes in response to major threats such as fishing, habitat degradation and climate change. This study used BRUVS to quantify and monitor elasmobranch abundances and distribution patterns in the Pacific of Costa Rica. Specifically, we (1) determined how elasmobranch assemblages were distributed across inshore and offshore sites of the Pacific of Costa Rica; and (2) investigated changes in species richness and abundance in relation to protection status (e.g. no-take vs. open-fishing sites), habitat composition and environmental drivers (e.g. water temperature and depth). These efforts led to the establishment of a national monitoring program focused on elasmobranchs and other key marine megafauna in Costa Rica, which may strengthen management and conservation approaches in the region.

## Results

**Figure 1:**
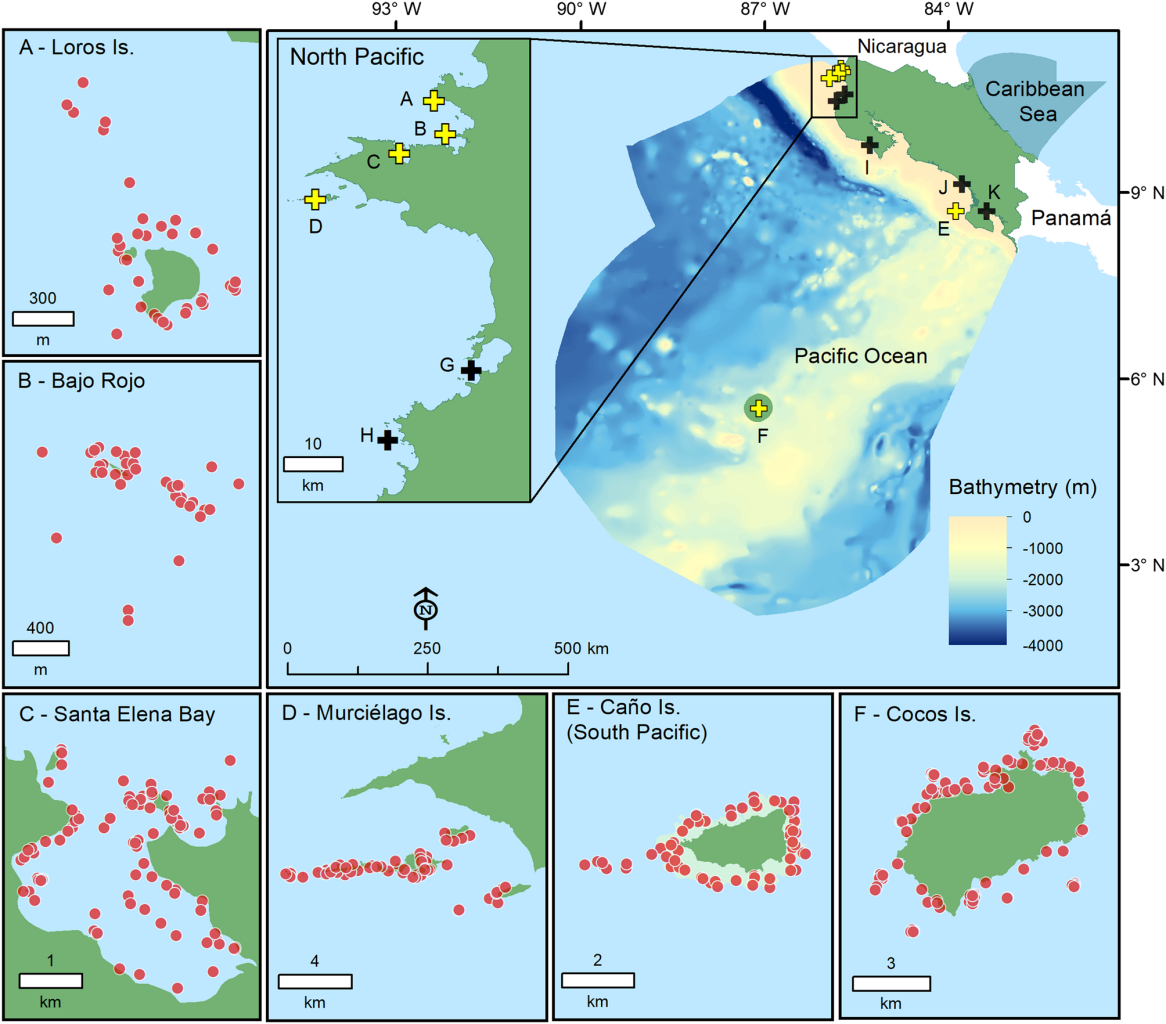
Map of Costa Rica (Central America) showing sampling sites selected to survey elasmobranchs. Color markers indicate the location of the main (yellow: **A**–**F**) and additional sampling sites (black: **G**–**K**). Red dots represent the location of baited remote underwater videos stations at the main sampling sites. Additional sampling sites: (**G**) Culebra Bay, (**H**) Flamingo, (**I**) Nicoya Península, (**J**) Marino-Ballena, (**K**) Golfo Dulce. The bathymetry within the economic exclusive zone is shown in the larger map. Sampling sites were plotted using base and raster layers from the Costa Rican Geographic Information System (GIS) Atlas open-access project (https://hdl.handle.net/2238/6749) in ArcMap 10.4 (ESRI, Redlands, California).

From the 430 BRUVS deployed across all sampling sites (Fig. 1), we detected a total of 29 elasmobranch species from 9 families (Table 1). Elasmobranchs were sighted in 87% of all BRUVS, and the number of species recorded per station varied from 1 to 7 (mean ± SD: 2.3 ± 1.3 species). The most commonly sighted species (i.e. species that were detected in more than 30% of the BRUVS deployed where they are known to occur) were the whitetip reef shark (*Triaenodon obesus*), the marble ray (*Taeniurops meyeni*), the Scalloped hammerhead (*Sphyrna lewini*) and the Galapagos shark (*Carcharhinus galapagensis*). Based on the different metrics of MaxN used to describe the relative abundance of elasmobranch species, the cownose ray (*Rhinoptera steindachneri*) (sum: 117; max = 76; mean ± SD: 10.6 ± 22.1), *S. lewini* (sum: 696; max = 70; mean ± SD: 7.4 ± 11.1) and *T. obesus* (sum: 674; max = 24; mean ± SD: 3.7 ± 3.5) were the most abundant elasmobranchs (Table 1). Species like *R. steindachneri* and *S. lewini*, for example, showed the maximum MaxN values, with 76 and 70 individuals detected on a single frame, respectively (Table 1). Based on the International Union for Conservation of Nature (IUCN) Red List, a large proportion of elasmobranch species recorded by BRUVS were threatened (66%).

**Table 1:** Summary of species recorded on baited remoted underwater video stations (BRUVS) in the Pacific waters of Costa Rica.

Family	Species	N	%N	MaxN			Rank	Status
				Mean ± SD	Max	Sum		
Carcharhinidae	*Triaenodon obesus*	183	61.8	3.7 ± 3.5	24	674	H	NT
	*Carcharhinus galapagensis*	51	33.6	1.6 ± 1	6	83	H	LC
	*Carcharhinus albimarginatus*	38	25.0	1.7 ± 1.6	8	65	M	VU
	*Galeocerdo cuvier*	59	19.7	1.2 ± 0.6	5	69	M	NT
	*Carcharhinus limbatus*	31	11.3	1.1 ± 0.4	3	35	M	NT
	*Carcharhinus melanopterus*	13	8.6	1.2 ± 0.4	2	15	L	NT
	*Carcharhinus falciformis*	10	6.6	1.2 ± 0.4	2	12	L	VU
	*Carcharhinus leucas*	8	5.7	1.2 ± 0.5	2	10	L	NT
Dasyatidae	*Taeniurops meyeni*	79	52.0	1.4 ± 0.8	5	109	H	VU
	*Hypanus longus*	61	22.8	1.5 ± 1	5	89	M	VU
	*Styracura pacifica*	1	0.4	1 ± NA	1	1	L	VU
Ginglymostomatidae	*Ginglymostoma unami*	60	22.9	1.4 ± 1.1	8	87	M	EN
Mobulidae	*Mobula birostris*	13	3.4	1.1 ± 0.3	2	14	L	VU
	*Mobula munkiana*	3	0.8	1 ± 0	1	3	L	VU
	*Mobula japanica*	1	0.3	2 ± NA	2	2	L	EN
	*Mobula tarapacana*	1	0.3	1 ± NA	1	1	L	EN
Myliobatidae	*Aetobatus laticeps*	65	15.7	1.4 ± 1.6	11	92	M	VU
Narcinidae	*Narcine entemedor*	3	4.5	1 ± 0	1	3	L	VU
	*Diplobatis ommata*	1	0.4	1 ± NA	1	1	L	VU
Rhinobatidae Rhincodontidae	*Rhincodon typus*	1	0.2	1 ± NA	1	1	L	EN
	*Pseudobatos prahli*	4	2.0	1 ± 0	1	4	L	VU
	*Zapteryx xyster*	3	1.4	1 ± 0	1	3	L	VU
	*Pseudobatos glaucostigma*	1	0.5	1 ± NA	1	1	L	VU
	*Pseudobatos planiceps*	1	0.5	1 ± NA	1	1	L	VU
Rhinopteridae	*Rhinoptera steindachneri*	11	2.7	10.6 ± 22.1	76	117	L	NT
Sphyrnidae	*Sphyrna lewini*	94	31.8	7.4 ± 11.1	70	696	H	CR
Urotrygonidae	*Urolophus halleri*	62	23.1	1.4 ± 0.9	5	89	M	LC
	*Urotrygon chilensis*	11	5.3	1.3 ± 0.5	2	14	L	NT
	*Urotrygon aspidura*	1	0.5	1 ± NA	1	1	L	NT

N—number of BRUVS with a species. Metrics of MaxN (maximum number of individuals of a species detected on a single frame) used to describe the relative abundance: mean ± SD; maximum (max); sum. Species were ranked based on their frequency of occurrence: *H* high (> 30% of the BRUVS); *M* medium (10–30% of the BRUVS); and *L* low (< 10% of the BRUVS). Conservation status (*DD* data deficient, *LC* least concern, *NT* near threatened, *VU* vulnerable, *EN* endangered, and *CR* critically endangered) based on IUCN Red List assessments^39^.

**Figure 2:**
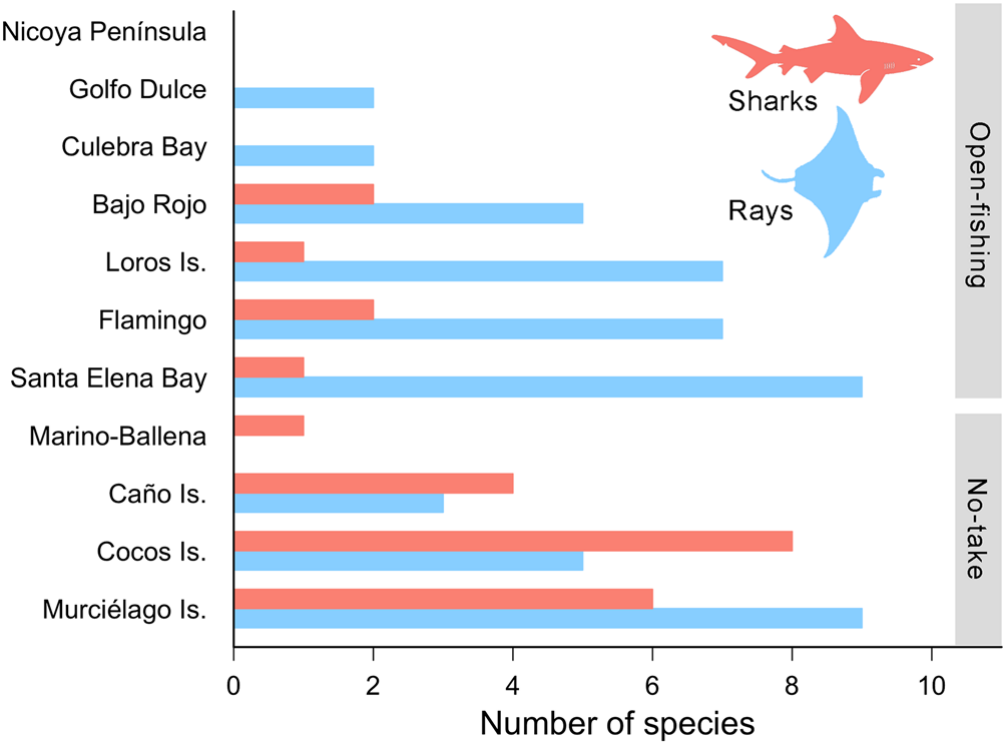
Number of elasmobranch species recorded with baited remote underwater video stations across all sampling sites in the Pacific waters of Costa Rica.

Elasmobranchs were detected at 10 of the 11 sampling sites (Fig. 1), with most sites except for Cocos and Caño Islands detecting a larger number of ray species than sharks (Fig. 2). However, only five coastal sites (Murciélago Islands, Santa Elena Bay, Loros Island, Bajo Rojo and Caño Island) and one offshore site (Cocos Island) were included in the analyses due to low sampling effort (sites with < 15 BRUVS; Table 2). The number of elasmobranch species recorded at these sites ranged from 7 species in Caño Island and Bajo Rojo to 15 species in Murciélago Islands, despite a four-fold increase in sampling effort at Cocos Island relative to coastal sites (Bajo Rojo and Loros Island; Table 2, Fig. 2). Based on the ECDF of species sightings, about 72–97% of all time to first sighting (TFS) events occurred by 75 min soak time across sites, and from 88 to 98% of recorded TFS events occurred by 90 min soak time across sites (Fig. S1). In Santa Elena Bay, Bajo Rojo and Caño Island, soak times of 75 min resulted in 72–82% TFS events, whereas the other sites ranged from 87 to 97%).

**Table 2:** Summary of sampling effort and elasmobranch species detected with baited remoted underwater video stations (BRUVS).

Protection status	Main sites	N	Effort (hr)	Elasmobranchs			%FF	%SP	%LP	%TS
				S	Richness	MaxN hr^−1^				
No-take	Cocos Is	158	231.2	11	3.2 ± 1.3	10.5 ± 9.8	16	26	58	85
No-take	Murciélago Is	67	115.6	15	1.6 ± 0.8	4.2 ± 10.4	14	57	29	70
No-take	Caño Is	59	113.9	7	1.4 ± 0.5	1.8 ± 1.2	14	43	43	31
Open	Santa Elena	42	86.3	9	1.8 ± 1.1	2.2 ± 1.5	11	78	11	69
Open	Loros Is	38	61.6	8	2.1 ± 1.0	3.7 ± 3.5	0	88	12	68
Open	Bajo Rojo	37	62.1	7	1.6 ± 0.9	2.0 ± 1.6	14	72	14	68

Protection status	Additional sites	N	Effort (hr)	Elasmobranchs			%FF	%SP	%LP	%TS
				S	Mean richness	MaxN hr^−1^				
Open	Flamingo	15	23.7	9	2.7 ± 1.6	3.4 ± 1.8	0	89	11	47
Open	Golfo Dulce	9	23.9	2	1.5 ± 0.7	2.5 ± 0.7	0	100	0	29
Open	Culebra Bay	3	3.5	2	1 ± 0	1 ± 0	0	100	0	67
No-take	Marino-Ballena	1	2	1	1	1	0	0	100	100
Open	Nicoya Península	1	1.5	0	0	0	0	0	0	0

Protection status: no-take and open to fishing. *N* number of BRUVS deployed, *S* number of species, *MaxN hr*^−1^ maximum number of individuals of a species recorded on a single frame standardized by soak time. *%FF* filter-feeders (species that feed on plankton), *%SP* small predators (species with average body sizes < 1.5 m total length), *%LP* large predators (species with average body sizes > 1.5 m total length), *%TS* threatened elasmobranchs based on current IUCN Red List assessments^39^. Data are reported as mean ± SD.

The proportion of elasmobranch species that were filter-feeders, small predators and large predators varied across sites (Table 2). A higher proportion of large predators were detected in not-take MPAs such as Cocos Island (58%), Caño Island (43%) and Murciélago Islands (30%). Sites with no protection status (Bajo Rojo, Loros Island and Santa Elena Bay) had a large proportion of small predators. Filter-feeders were recorded opportunistically across sites (Table 2). In addition, more than half of elasmobranch species recorded at the main sampling sites were threatened (> 60%), except for Caño Island (31%).

**Figure 3:**
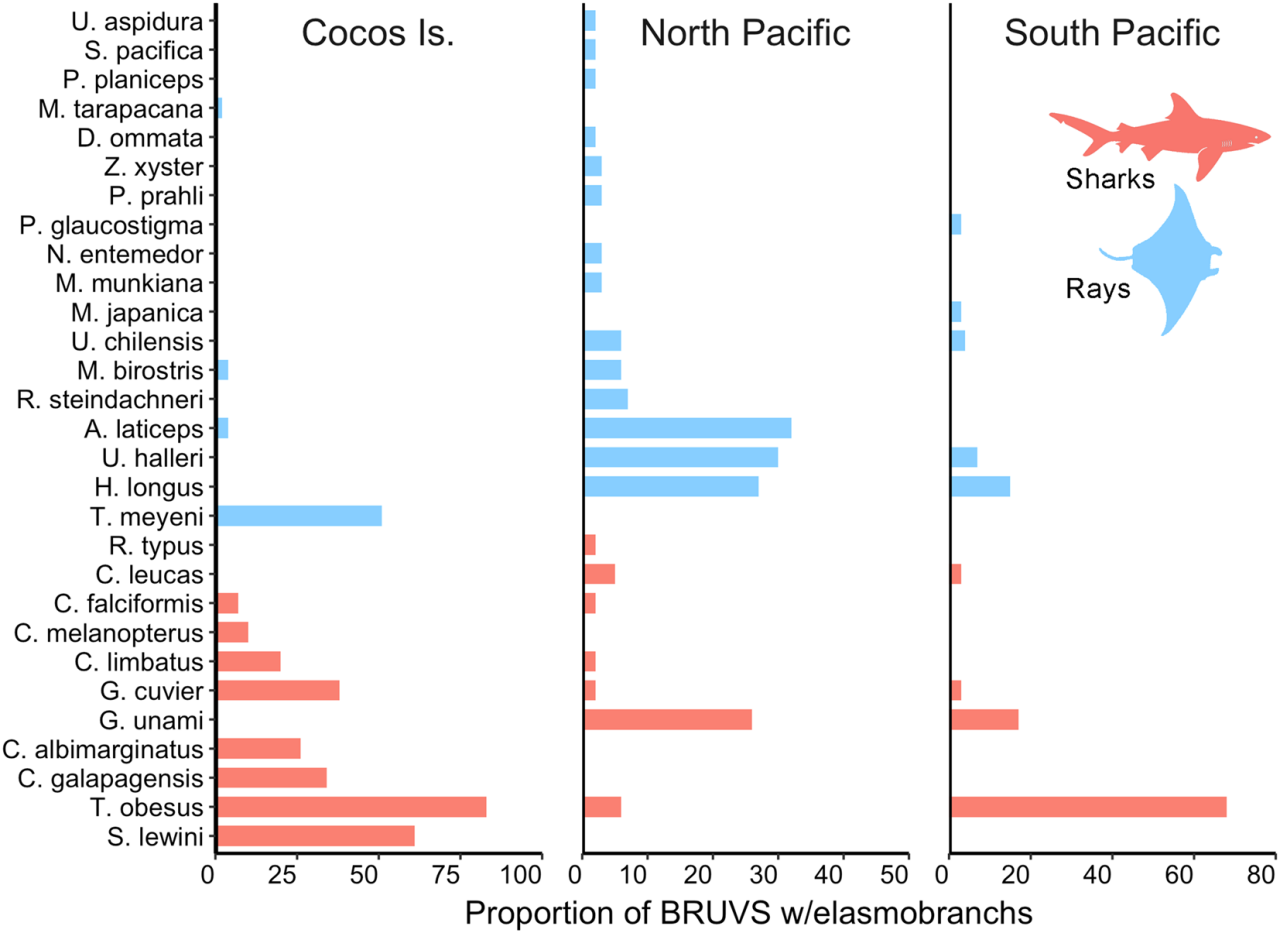
Proportion of baited remote underwater video stations (BRUVS) that recorded elasmobranch species in different regions from Pacific waters of Costa Rica.

Overall, BRUVS recorded more elasmobranchs in the North Pacific (21 species) than in Cocos Island (12 species) and the South Pacific (9 species) regions (Fig. 3). However, we also deployed more BRUVS in the North Pacific (N = 203) relative to other regions (Cocos Island: N = 158; South Pacific: N = 69). The frequency of occurrence also varied among regions, with all BRUVS deployed at Cocos Island detecting at least a single elasmobranch species. In the North and South Pacific regions, the frequency of occurrence was 78% and 84%, respectively.

### Elasmobranch assemblages across sites

**Figure 4:**
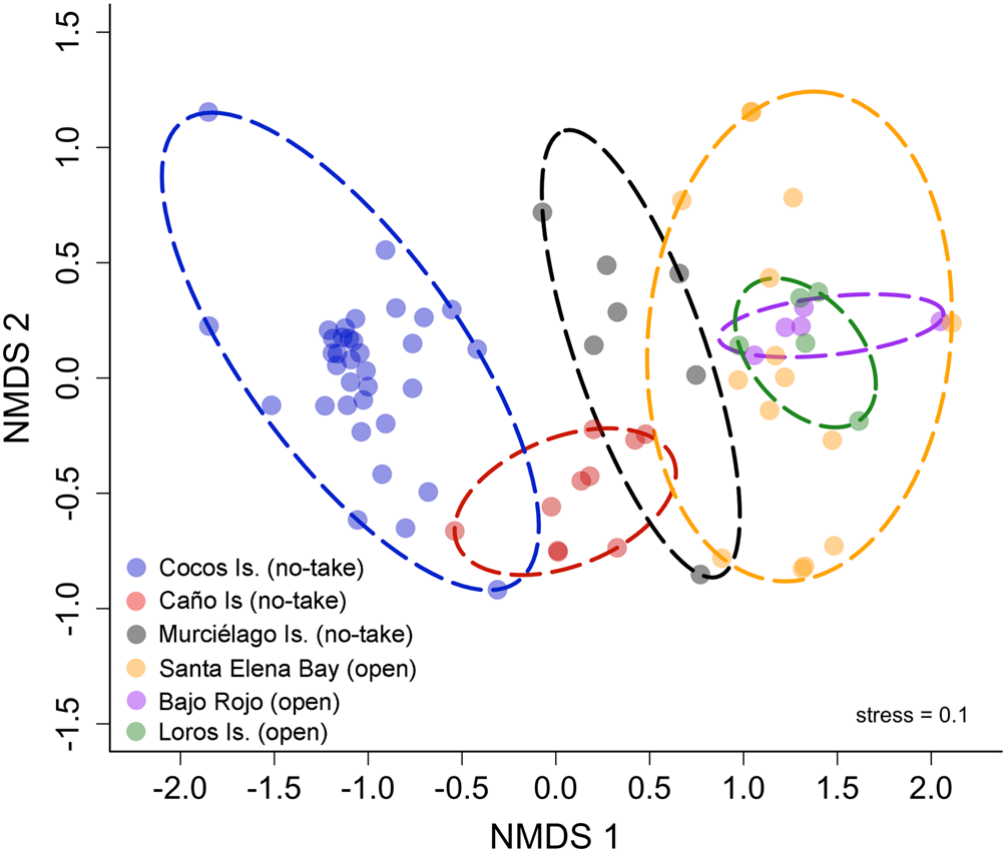
Non-parametric MDS plot showing the separation of elasmobranch assemblages among no-take and open-fishing sites in Pacific waters of Costa Rica.

**Figure 5:**
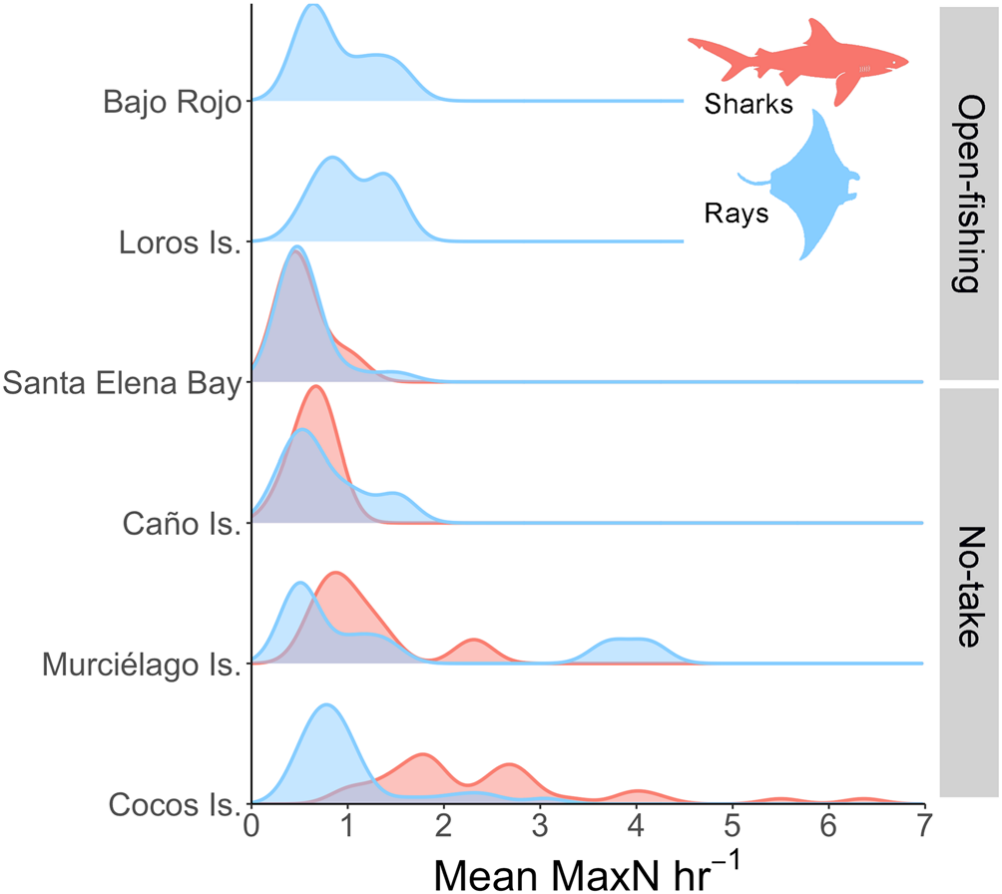
Frequency curves showing the mean relative abundance (MaxN hr^−1^) of sharks and rays recorded using baited remote underwater video stations at six sites from the Pacific of Costa Rica. The relative abundance of elasmobranchs sighted at each station was average across sites and sampling dates.

**Figure 6:**
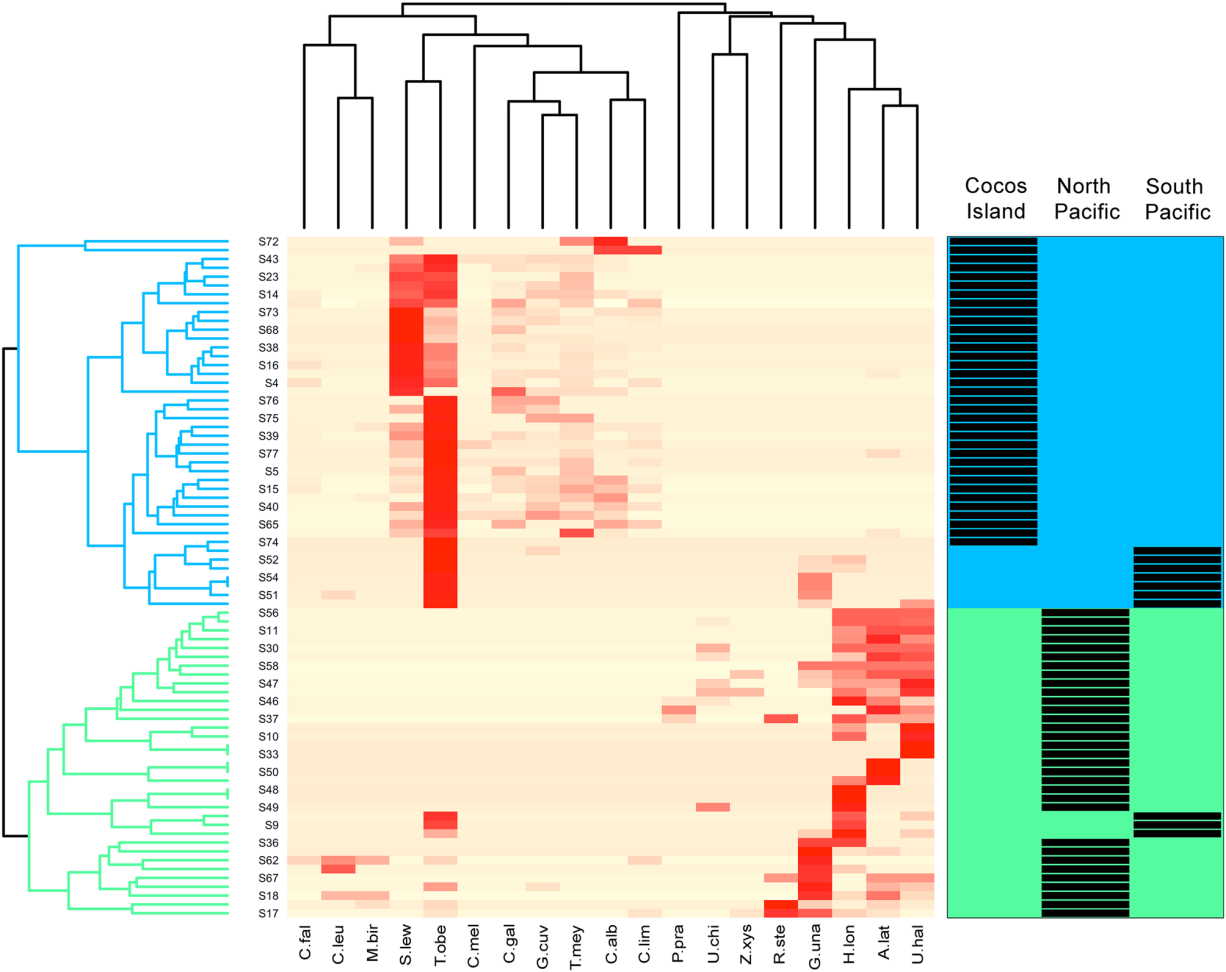
A heatmap with dendrograms showing hierarchical clustering of elasmobranch relative abundance (MaxN hr^−1^) by site (row samples) and by the level of species co-occurrence (column samples). A species-site matrix was constructed to examine how the samples (i.e. BRUVS deployed on the same site and date were pooled together and treated as single independent samples) clustered by site. Ten species were removed from the analysis (*Diplobatis ommata*, *Narcine entemedor*, *Styracura pacifica*, *Urotrygon aspidura*, *Mobula Japonica*, M. *munkiana*, *M. tarapacana*, *Rhincodon typus*, Pseudobatos glauca, *P. planiceps*) due to low frequency of occurrence. Heat map allows identifying dominant species based on their relative abundance (more intense red colors). Samples were further classified by region (right-side plot) and the two main resulting clusters (green and blue colors) are highlighted.

Differences in elasmobranch assemblages were detected among sites (F_5,65_ = 21.3, *p* < 0.001; R^2^ = 0.64). Pairwise comparisons revealed that elasmobranch assemblages from no-take MPAs (Cocos Island, Caño Island and Murciélago Island) were significantly different from each other and were also different from open-fishing sites (Bajo Rojo, Loros Island and Santa Elena Bay), which shared similar assemblages (Fig. 4). Relative abundance of elasmobranchs also differed among sites, but mean MaxN hr^−1^ of rays was higher than sharks in open-fishing sites relative to no-take MPAs, particularly for Cocos and Murciélago islands (Fig. 5). The cluster analysis revealed a significant separation of samples by region, with all samples from Cocos Island and the North Pacific region forming two distinct clusters (Fig. 6). Interestingly, most of the samples from Caño Island clustered together with samples from Cocos Island; only a few samples shared more similarities with the North Pacific. In addition, in many of the samples from the South Pacific (e.g. Caño Island), BRUVS recorded a high abundance of *T. obesus* and at least one tiger shark (*Galeocerdo cuvier*), which are both dominant species in Cocos Island. The heatmap also revealed that *S. lewini* and *T. obesus* tend to be detected in the same stations of Cocos Island, whereas coastal batoids such as the longtail stingray (*Hypanus longus*), the round stingray (*Urobatis halleri*) and *A. laticeps* co-occurred at stations from the North Pacific (Fig. 6).

### Drivers of elasmobranch abundance and diversity

**Figure 7:**
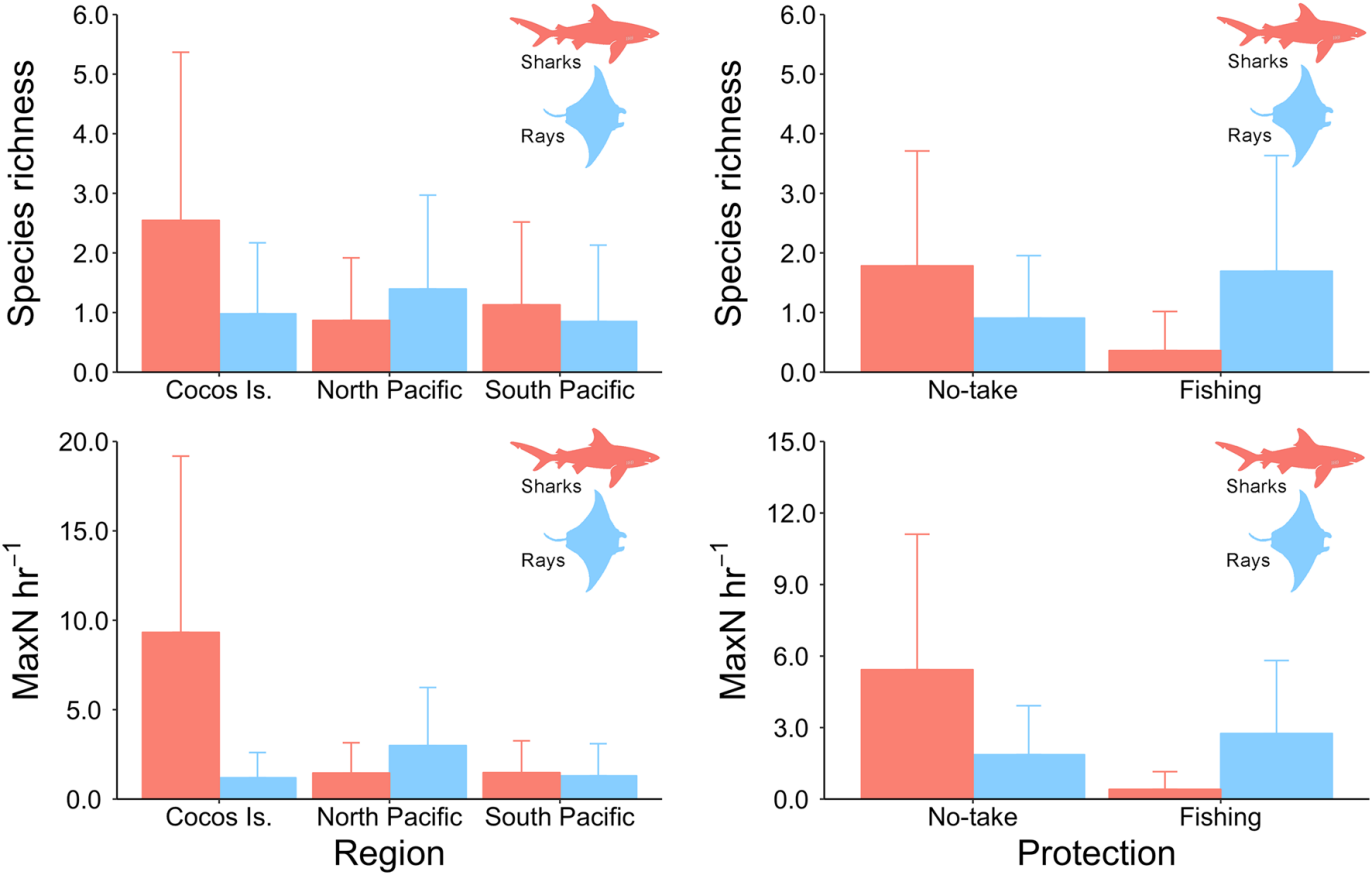
Predictions of elasmobranch species richness and relative abundance (MaxN hr^-1^) by region and habitat protection based on Poisson generalized linear models. Error bars represent the 95% confidence intervals.

Based on maximum likelihood ratio tests and AIC, the best fitted GLM model that explained elasmobranch species richness included region, habitat protection, taxonomical group and depth (Table S1). The best fitted model that explained the relative abundance (MaxN hr^−1^) of elasmobranchs included the habitat PC2 scores (rock/turf to sand/rubble), in addition to the same predictors that explained elasmobranch richness (Table S1). Poisson GLM models revealed significant interactions between region × elasmobranch group and protection × elasmobranch group for both species’ richness and abundance (Table 3). Overall, there was a significantly higher shark diversity and relative abundance at Cocos Island relative to the other regions. In contrast, ray diversity and relative abundance was significantly higher in the North Pacific region (Fig. 7). In the South Pacific, BRUVS recorded a similar species richness and abundance for both sharks and rays. Habitat protection also had an effect on elasmobranch species richness and abundance, but only shark species seemed to benefit from MPAs, whereas the diversity and abundance of rays was significantly higher at open fishing sites (Table 3, Fig. 7). Depth had a significant positive effect on elasmobranch richness and abundance, whereas an increase in the PC2 scores partially explained a greater elasmobranch abundance (Table 3).

**Table 3:** Summary results of Poisson generalized linear models (GLM) used to examine the effect of different drivers on elasmobranch species richness and relative abundance (MaxN hr^-1^).

	DF	Deviance	Resid. Df	Resid. Dev	*p* value
**Species richness**					
Full model			428	651.55	
Region	2	112.46	426	539.09	< 0.001
Protection	1	4.333	425	534.76	0.037
Group	1	25.891	424	508.87	< 0.001
Depth	1	11.828	423	497.04	< 0.001
Region × group	2	81.256	421	415.78	< 0.001
Protection × group	1	29.765	420	386.02	< 0.001
**Relative abundance**					
Full model			428	5144.9	
Region	2	1181.09	426	3963.8	< 0.001
Protection	1	20.92	425	3942.9	< 0.001
Group	1	330.01	424	3612.9	< 0.001
Depth	1	92.21	423	3520.6	< 0.001
PC2 (rock/turf to sand/rubble)	1	19.95	422	3500.7	< 0.001
Region × group	2	746.84	420	2753.9	< 0.001
Protection × group	1	16.74	419	2737.1	< 0.001

**Figure 8:**
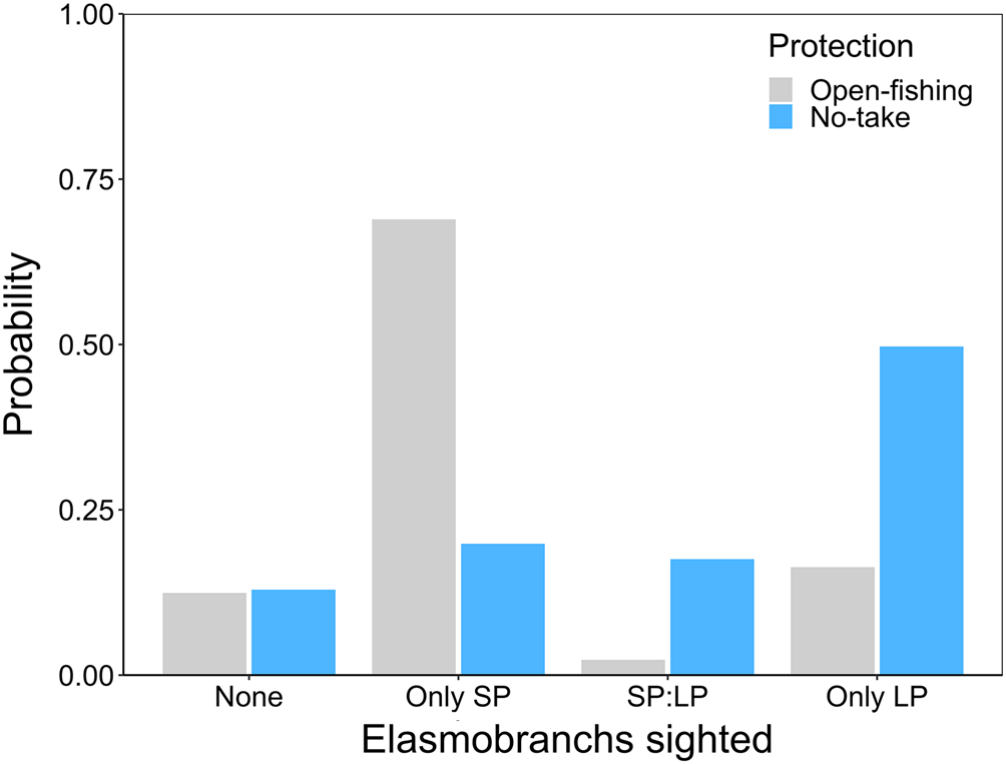
Predicted probability of elasmobranch sightings estimated from baited remoted underwater video stations in no-take and open-fishing sites. *SP* small predators, *LP* large predators, *SP:LP* equal proportion of SP and LP.

An ordinal logistic regression model showed that habitat protection had a significant effect on the number of BRUVS that recorded small or large predatory elasmobranchs (t value = 10.3, *p* < 0.0001). In open-fishing sites, there was a significantly higher probability (70%) of detecting only small predators, whereas in no-take sites the probability of detecting an equal proportion of small/large predators or only large predators was 18% and 50%, respectively (Fig. 8). Moreover, there was a low probability (15%) of detecting small predators in no-take sites.

## Discussion

This study adds on previous evidence that BRUVS are capable of providing fast and robust estimates of the distribution, abundance and diversity of elasmobranchs over large spatial and temporal scales^19,21^, information that is crucial by managers to detect population-level changes in response to multiple threats such as overfishing, habitat degradation and climate change^4,6^. Based on our findings, BRUVS were able to detect approximately 31% of the entire elasmobranch diversity reported for Costa Rica^27^. Although our study surveyed a wide variety of coastal and offshore habitats where elasmobranchs are known to occur, due to logistic and budgetary reasons, our sampling was restricted to shallow benthic habitats (< 60 m deep) from the Pacific. When excluding Caribbean elasmobranchs, as well as fully pelagic and bathyal species, our benthic and mid-water BRUVS detected 54% of the entire elasmobranch diversity. Moreover, our data revealed that while large predators can benefit from no-take MPAs, they were relatively uncommon or completely absent from open-fishing sites.

In the Pacific of Costa Rica, elasmobranch assemblages have been previously described using a wide range of fishery-dependent and independent methods^27^. However, with the exception of a few fishery-dependent studies that surveyed elasmobranchs over the entire continental shelf and economic exclusive zone (EEZ) of Costa Rica^16,35,40^, most studies have been restricted to relatively small areas, including Cocos Island^41,42^, Golfo Dulce in the South Pacific^43^, and Tárcoles in the Central Pacific^44^. Fishery surveys usually provide valuable information on elasmobranch assemblages and their distribution, particularly for species that are harder to detect using traditional survey approaches^45,46^. For example^16^, found 24 elasmobranch species from 9 families associated to the longline fishery, whereas^33^ found 25 demersal elasmobranchs from 14 families in the trawling fishery. Both of these studies reported similar species richness, but different composition compared to our study (Table 4, Table S2). Although most families captured by long-line and trawling were detected in the current study (Table 4), only nine of these species were present across studies (Table S2). The rest were either obligate pelagic or demersal species that are rare or absent from shallow reef habitats (Table S2)^27^. While a combination of BRUVS and fishery-dependent surveys can detect elasmobranch species on a wide range of habitats, extractive fishing techniques alone suffer from gear-specific sampling bias, are usually not suitable in hard bottoms, are limited to open-fishing areas or may be detrimental to the survival of threatened species^14,47^.

**Table 4:** Studies of elasmobranchs recorded in the Pacific of Costa Rica using different methods.

Family	Trawling^35^	Submarine^41^	Longline^16^	Diving^42^	Diving^48^	This study
Alopiidae	–	–	2	–	–	–
Carcharhinidae	1	1	8	6	1	8
Dasyatidae	1	2	2	1	2	2
Echinorhinidae	1	1	–	–	–	–
Ginglymostomatidae	–	–	1	–	1	1
Gymnuridae	1	–	–	–	–	–
Mobulidae		2	3	2	–	4
Myliobatidae	2	1	1	1	–	2
Narcinidae	3	–	–	–	–	2
Odontaspidae	–	1	–	–	–	–
Potamotrygonidae	–	–	–	–	–	1
Pseudocarcharhiidae	–	–	1	–	–	–
Rajiidae	3	–	–	–	–	–
Rhincodontidae	–	1	–	1	1	1
Rhinobatidae	1	–	–	–	–	3
Squatinidae	1	–	–	–	–	–
Sphyrnidae	1	1	4	1	–	1
Torpedinidae	1	1	–	–	–	–
Triakidae	2	–	2	–	–	–
Trygonorrhinidae	1	–	–	–	–	1
Urotrygonidae	6	–	–	–	–	3
Number of species	25	11	24	12	5	28

Elasmobranch studies in Costa Rica using fishery-independent methods have also reported similar species richness and composition compared to our study, at least at local scales (Table 4, Table S2). For example^39^, used data collected by divers over 21 years to investigate elasmobranch population trends at Cocos Island, whereas^38^ analyzed video footage from submarine surveys between 2006 and 2012 to examine elasmobranch assemblages in deep waters. All the species recorded by^39^ were also detected in our study (Table S2). However, due to the capability of the submarine to survey deeper waters (up to 330 m)^38^, reported four elasmobranch species that were not detected by our BRUVS (Table S2). Our study also reported more elasmobranch species at Caño Island than traditional underwater visual surveys^48^, which are often biased by divers’ experience and limited by time, depth and weather conditions^15^. In contrast, BRUVS provide a relatively cost-effective survey method that enhances predator detection by using bait and overcomes fish avoidance issues with divers^25,49,50^. Moreover, permanent footage generated through BRUVS can be used to answer specific behavioral questions^51^ or shared with other researchers to support regional and global conservation efforts such as the one lead by the Global FinPrint Project^22^.

Few studies have used BRUVS to assess shark assemblages in the ETP region, and those were restricted to remote and isolated islands^52,53^. For example^52^, reported 10 species of sharks from 4 families in the Galapagos archipelago (Ecuador), whereas^53^ detected 8 species of sharks from 2 families in Revillagigedo Islands (Mexico). Similar trends in terms of species composition were observed in Cocos Island, in which our BRUVS were able to detect 8 species of sharks from 2 families. However, sampling effort at Cocos Island (158 BRUVS) was considerably lower than in the Galapagos archipelago (629 BRUVS) and relatively similar to Revillagigedo Islands (112 BRUVS deployed). Cocos Island is also 22 and 78 times smaller than Revillagigedo and Galapagos Islands, respectively. The Galapagos archipelago also has some species from the family Triakidae and Heterodontidae that are absent from Cocos and Revillagigedo Islands^52^. These findings further highlight the efficiency of using BRUVS to survey and monitor elasmobranch species at remote and isolated islands from the ETP, some of which are recognized as UNESCO World Heritage sites.

Numerous studies have demonstrated that shark abundances are generally greater inside than outside not-take MPAs^19,22,54^, which is in agreement with our findings. However, BRUVS deployed at Caño and Murciélago islands had significantly less species and lower abundances compared to Cocos Island. Differences among MPAs may be related to size, human accessibility and levels of enforcement, which have been previously identified as key drivers of MPA efficiency towards elasmobranch protection^55–57^. Cocos Island, for example, is a relatively pristine, remote and diverse island that currently has one of the largest fish biomass in the Eastern Tropical Pacific^58,59^. Moreover, given its status as a UNESCO World Heritage Site, more resources for surveillance and enforcement are allocated to Cocos Island than any other MPA in Costa Rica. In contrast, Caño and Murciélago are smaller inshore islands that are in close proximity to important fishing towns, and have limited resources and personnel, which results in higher levels of illegal fishing^60^.

Illegal fishing is a major threat affecting elasmobranchs, even in remote protected areas such as Cocos Island, where ensuring compliance is challenging^30,61,62^. Moreover, the increase in foreign pelagic fleets with higher fishing capacity and poor landing statistics remain an ongoing issue in the EEZ of Costa Rica^28,63^, and may be linked to recent population declines of threatened pelagic species in the ETP^42^. Therefore, despite the relative pristine conditions of Cocos Island, better management strategies for highly mobile elasmobranch species that move beyond reserve boundaries are necessary to allow population recovery and more effective protection schemes^62,64^.

Anthropogenic impacts such as habitat loss and degradation represent additional threats in coastal areas, which are generally exposed to multiple chronic stressors^56,65^, and therefore, may be important drivers of elasmobranch population declines^4,38^. Efforts towards monitoring key coastal habitats for elasmobranchs are needed, as some of these may also function as critical habitats (e.g. nursery, reproductive and/or feeding grounds) for threatened species^36,66,67^. In Costa Rica, rapid coastal development and changes in land use practices are already impacting the health of coral reefs and reef fish communities in some areas^68,69^. However, despite their close proximity to human centers and limited resources for surveillance, Caño and Murciélago islands are still two of the healthiest and most productive areas in Costa Rica, and provide numerous ecosystem services for coastal communities^48,70,71^.

The absence of large predators from marine habitats is often considered an indicator of fishing pressure^72,73^, while their presence may be critical to restoring or maintaining ecosystem function and health^74,75^. Based on our findings, the proportion of large predators (> 1.5 m total length) was greater inside no-take sites, whereas small predators were more diverse and dominated the elasmobranch assemblage in open-fishing sites. Moreover, larger species that may be defined as true apex predators such as the tiger (*Galeocerdo cuvier*), bull (*Carcharhinus leucas*), silky (*C. falciformis*) and Galapagos (*C. galapagensis*) sharks were exclusively detected inside coastal and offshore no-take reserves. All open-fishing sites included in this study (Loros Island, Bajo Rojo and Santa Elena Bay) were located in the North Pacific of Costa Rica, an area that has been historically exposed to strong pressure from semi-industrial and artisanal fisheries^28,76^. Declining shark populations since the 1990s have likely resulted in changes in fish community structure and composition, as well as less productive and more degraded habitats^69,70^. Furthermore, anecdotal information from local stakeholders indicate that apex predators (e.g. *S. lewini*, *C. leucas*, *C. limbatus* and *G. cuvier*) were often captured in gillnets and coastal long-lines in the late 1980s and early 1990s, but currently are uncommon in open-fishing sites from the North Pacific (Lara. A. & Lara. M., pers. comm).

The decline of large sharks from coastal habitats in Costa Rica may explain why ray diversity and abundance peaked in open-fishing sites. This observation is consistent with the hypothesis of “mesopredator release”, where removal of top predators may propagate down the food web leading to an increase in the abundance of mesopredators, a process that can have negative ecological and economic consequences at the ecosystem level^22,77^. Predators can also induce habitat shifts in prey that rely on crypsis and refuging^51^, which may explain common interactions between some rays (e.g. *Urobatis halleri* and *Hypanus longus*) and the BRUVS at open-fishing sites. Although there is some evidence that the absence or decline of top predators from coastal habitats in Costa Rica may result in shifts in community structure and function^69^, more studies are still needed to further elucidate major drivers influencing these patterns. Increasing the number of BRUVS deployed in no-take and open-fishing sites from the Central and South Pacific regions may help clarify whether fishing and/or other drivers are responsible of the shifts in predator abundance.

Environmental factors such as temperature, depth and habitat composition are also key drivers of elasmobranch assemblages^31,78,79^, but these drivers had little or no contribution in our study. Based on our results, there was a positive effect of depth on both elasmobranch species richness and abundance, which is consistent with findings from other studies^19,80^. Changes in temperature, light level and productivity associated to depth may explain some of the observed trends in elasmobranch assemblages^78^. For example, Scalloped hammerhead sharks have been shown to select cooler temperatures below the thermocline^81^. Due to logistic limitations associated with strong trade winds, we could not survey Murciélago Island during the upwelling season. Therefore, the temperature range between surveyed sites was probably not wide enough to observe differences among elasmobranch assemblages. Greater seasonal replication at each site (at least twice per year to cover dry and rainy seasons) would provide the possibility to assess seasonal variation and the effect of periodic climate events such as ENSO on elasmobranch assemblages. Furthermore, implementing BRUVS deeper than 60 m (maximum depth recorded in this study) would probably increase the number of species detected and provide valuable data about changes on species compositions with wider depth gradients. The effect of habitat and other environmental drivers such as productivity, oceanographic conditions and prey availability on elasmobranch assemblages should be further investigated at the site level to better understand potential anthropogenic impacts.

Overfishing has been identified as the most significant and widespread threat affecting elasmobranch species at a national^27^ and global scales^1,4,5^. Some efforts made in Costa Rica within national and coastal waters, are crucial at reducing fishing impacts on elasmobranch populations, and could potentially lead to their recovery. For example, a recent partnership between the government of Costa Rica and the private sector led to the installation of a marine radar in Cocos Island in 2016, which has proven to be instrumental to achieve a better control and surveillance of fishing activities in the island. However, conservation actions and more effective management are still needed to reduce fishery impacts on threatened elasmobranchs that use the EEZ in their migratory route between oceanic islands of the ETP and the mainland^11,37,81^. In addition, in recent years the use of coastal gillnets in the North Pacific region has been largely reduced (M. Lara comm. pers), and since 2013, the government of Costa Rica stopped issuing new trawl fishing licenses; the last one expired in August 2019. Moreover, Costa Rica has a network of 20 MPAs, which protect approximately 17.5% of national waters^60^, but only 0.9% of the EEZ, where fishing pressure remains high^16^. Therefore, in order to achieve elasmobranch conservation goals, it is necessary to reduce fisheries bycatch, improve landing statistics, increase the level of enforcement and resources allocated to current MPAs, and in some cases apply the precautionary approach^27^.

Recent IUCN assessments of pelagic and endemic species from the ETP revealed that the number of threatened elasmobranchs in Costa Rica increased from 17^27^ to 53% (43 species)^39^. These alarming statistics demonstrated that most elasmobranch species that were “Data Deficient” are now listed as threatened when more information became available^4^. Moreover, some species that were highly abundant in our surveys due to their schooling behavior such as *S. lewini* changed from Endangered to Critically Endangered based on recent assessments by the IUCN Red List^37^. Our study showed that over half of the species detected by BRUVS (66%) were threatened^39^; therefore, a well-designed BRUVS survey may provide crucial information on relative abundance trends for assessing the conservation status of elasmobranchs.

BRUVS have been widely recognized as a suitable sampling method to answer key questions on elasmobranchs^19,22,51^; however there are some biases associated that are important to recognize. For example, BRUVS are less effective in turbid waters with low visibility, they attract large predators, which may reduce detectability of smaller species, their bait plume dispersion is unknown, and thus our knowledge of the sampling area, and they tend to have a restricted field of view compared to observers in the water^14^. Another limitation from our study was that smaller fishes were able to take some of the bait from the containers, thus reducing the effect of the bait plume^82^. Our study is mainly focused on predator species, therefore, the use of bait with its associated biases is necessary to increase detectability of cryptic and threatened species with low natural abundances^25^. Furthermore, video samples with low visibility were not considered in the analysis overcoming turbidity biases associated with BRUVS. Finally, our survey inside MPAs required a non-destructive approach like BRUVS, thus the advantages of this technique may outweigh the limitations.

To our knowledge, this is the first study using BRUVS that provides a detailed assessment of elasmobranch distribution and abundances in both coastal and offshore sites from the ETP region. With considerably less sampling effort than traditional fishery-dependent and independent methods, our study was able to survey key coastal and offshore sites from Pacific waters of Costa Rica, and recorded a greater number of elasmobranch species. Comparisons with other studies in the ETP further recognize the value of using BRUVS as an accessible technique capable of overcoming the logistical constraints of long-term surveys for elasmobranch populations in the region.

Our results further demonstrate the benefits of no-take MPAs for large predators and confirm the negative impacts of humans on elasmobranch assemblages in near coastal habitats. However, some economically important or threatened species that are generally found in fully pelagic or deep-water demersal habitats were not detected by the BRUVS, which are often an important target of trawling and long-line fisheries^27^. Therefore, we recommend that further surveys should also include the use of deep-water benthic and pelagic-BRUVS to achieve a more comprehensive assessment of key habitats for elasmobranch species, as well as for identifying potential connectivity routes between oceanic islands and coastal habitats^83^.

This study is currently being used a reference baseline for developing a national monitoring protocol of elasmobranchs and key marine megafauna in Costa Rica. A standardized methodology using BRUVS is presented in a logistically and economically feasible protocol that promotes the participation of park-rangers in field work, data processing, video analysis and data interpretation. By doing so, we reduce the costs of fieldwork and promote the long-term application of the protocol by stake-holders. The conservation status of elasmobranchs and other large pelagic fishes included as focal species in the protocol is evaluated through a set of indicators in order to detect short term changes and provide early alerts that translate into faster management actions by decision makers. This protocol was first tested at Cocos Island National Park, and subsequently adopted by other MPAs in Costa Rica. A summary of the proposed standards regarding experimental design using BRUVS is presented at Table S3 and Table S4. Although we acknowledge that ideal sampling designs depend on specific research questions, habitats and species surveyed^84^, here we provide general guidelines to standardize BRUVS surveys on reef-associated elasmobranch populations in tropical marine ecosystems. The use and optimization of this protocol by other countries, especially in Latin America could significantly increase the limited ecological data available for many threatened and/or migratory species leading to more effective management and conservation approaches at the regional level.

## Methods

### Sampling sites

This study was conducted at 10 coastal sites from the continental shelf of Costa Rica and in Cocos Island, an oceanic island located approximately 500 km southwest from the mainland (Fig. 1). Coastal sites were distributed in the North and South Pacific regions, and included a wide range of structurally complex and diverse habitats (e.g. rocky/coral reefs, sandy/muddy bottoms and macroalgae) from near-shore islands to small islets, underwater pinnacles, and a large bay. We classified the 11 sampling sites into “main study sites” (those sites with > 15 BRUVS deployed) and “additional sites” (< 15 BRUVS deployed) (Table S5). Additional sites were only used to summarized general patterns of species richness across regions.

In the North Pacific, we sampled four main sites (Loros Island, Bajo Rojo, Santa Elena Bay and Murciélago Islands) across a stretch of ocean of 65 km, approximately 1–6 km off the coast. All of these sites are located in the Guanacaste Marine Conservation Area (ACG), a region that is known for its high biodiversity and coastal productivity^60,86^. Murciélago Islands consist of five small islands and ten islets which represent the largest group of coastal islands in Costa Rica and the only area that we sampled in the North Pacific region that has been fully protected since 1987 (463.91 km^2^)^48,60^. Santa Elena Bay has an area of approximately 7.3 km^2^, and contains a variety of critical habitats for fish, including coral and rocky reefs, mudflats, sandy bottoms, mangroves, and estuaries^87^. Santa Elena Bay was declared a marine management zone in 2018, where only sport and artisanal hook-and-line fishing are allowed in some areas of the bay. Isla Loros and Bajo Rojo are areas completely open to sport and artisanal fishing activities, including hook-line and compressor fishing. Coral diversity and distribution in the North Pacific region is influenced by a seasonal upwelling event between December and April, combined with strong wave action^87,88^. The upwelling brings cool nutrient-rich water from the bottom, enhancing coastal productivity and biodiversity^87^. During the upwelling, water temperature can drop from an annual average of 30–15 °C^88,89^.

In the South Pacific region, we sampled Caño Island, a 2.9 km^2^ island located 15 km off the coast that was declared a marine reserve (55.3 km^2^) in 1987^60^. The island has one of the most diverse coral reef formations in the Pacific south of Costa Rica ^90,91^ characterized by five fringing coral reef flats ranging in size from 0.008 to 0.042 km^2^, covered by crustose coralline algae, isolated live colonies of pocilloporids and poritids, and microatolls of massive coral *Porites lobata*^92^. The shallow sections of the reef are structured mainly by physical factors (e.g. wave action, temperature and salinity fluctuations, and low tide exposure), whereas the deeper sections are influenced by biological interactions such as bioerosion, damselfish algal lawns, and corallivores^92^.

Cocos Island is an oceanic island located 500 km southwest of mainland Costa Rica (5°52′N, 87°06′W) (Fig. 1). Given the high level of endemism and biodiversity, Cocos Island was declared a National Park in 1978 and an UNESCO World Heritage site in 1997. The MPA has a surface area of approximately 2011 km^2^^60^, and has one of the largest fish biomasses in the tropics^58,59,93^. Cocos Island has a complex bottom morphology^94^, with a high variety of habitats including, sandy and rocky bottoms at various depths and extensive coral reefs mainly found at the north side of the island inside two of the largest bays along the coast^86,95^. The southern side of the island is more exposed to the currents and waves typically coming from the southwest^96^ and is characterized mainly by rocks covered in barnacles and other invertebrates^97^. This oceanic island has a marked seasonality due to the influence of the North Equatorial Countercurrent (NECC), the main west–east current near the equator^96,98^. During the rainy season (July–November), the effect of the NECC is enhanced, promoting more productivity, lower temperature and stronger currents^98^. Sea surface temperatures at Isla del Coco range from 24 to 29 °C and are affected every 4–9 years by the ENSO, which can result in temperatures of up to 30.9 °C^34^.

### Sampling design

We used BRUVS to quantify the distribution and abundance of elasmobranch species across a wide range of habitats and depths, following a biologically informed stratification of sites and replicates along the Pacific of Costa Rica. Sampling sites in this study were selected based on the protection status, reef/rocky formations where species may aggregate or because they represented a gradient of fishing intensity and habitat degradation. Within each site, we tried to cover the main habitats available (e.g. coral and rocky reefs, sandy bottoms, rhodolith beds, mangroves, etc.) based on previous studies, local ecological knowledge and guidance from Park Rangers when surveying some of the conservation areas of Costa Rica. Two types of BRUVS were used according to the bottom structure at each surveyed habitat: (1) benthic BRUVS – a pyramid-shaped steel frame that was lying on the seabed (for a graphical representation of the design see Ref.^99^); and (2) mid-water BRUVS—a triangle-shape steel frame that had a small weight anchored to the bottom and was suspended 1–2 m from the seafloor in the water column by an underwater buoy system (for a graphical representation of the design see Ref.^52^). Benthic BRUVS were used to record species associated with flat or less irregular seabed, whereas mid-water BRUVS were used to record species around topographically complex structures (e.g. underwater pinnacles) or more irregular bottoms in order to maximize the quality of the video files and BRUVS retrieval. Both BRUVS designs had detachable bait arms, consisting of a metal bar and a PVC mesh cylinder containing 1–1.5 kg of crushed mackerel (*Scomber japonicus*), a locally sourced bait used by the longline fishery of Costa Rica. Polystyrene surface floats were attached by an 8 mm polypropylene rope to facilitate retrieval of steel frames from small boats. Each BRUVS had a single camera (GoPro Hero4) with additional battery pack and was set to record at a rate of 60 frames per second/1080p resolution. BRUVS were deployed during daylight hours (8:00–17:00) at depths ranging from 1 to 60 m (mean ± SD; 15.04 ± 8.9 m) and set approximately 300–500 m apart from each other to ensure sample independence^19,99^. Soak times (effective time when BRUVS were recording at the bottom) for all deployments varied from 50 to 200 min (mean ± SD: 103 ± 29 min) and BRUVS deployments per site from 37 to 158 (Table S5). Water visibility was generally above 5 m and up to 20 m at Cocos Island, but some of the coastal sites had poor visibility (< 5 m) and consequently were removed from the analyses. A temperature datalogger (ONSET Hobo Pendant) was attached to the steel frame to monitor water temperature at 1-min intervals. We also recorded the unique BRUVS ID (combination of the station No. + GoPro No. + site), location (latitude/longitude), date/time and depth. Sampling sites were plotted using base and raster layers from the Costa Rican Geographic Information System (GIS) Atlas open-access project (https://hdl.handle.net/2238/6749) in ArcMap 10.4 (ESRI, Redlands, California) (Fig. 1).

Video footage was processed and analyzed using the software EventMeasure (SeaGIS) in order to: (1) manage data from field operations and video reading; (2) capture the timing of events; and (3) capture reference images of the seafloor and sharks in the field of view. To avoid recounting individuals, we used the MaxN (i.e. maximum number of individuals from each species observed in a single video frame) as a conservative measure of relative abundance^14^. All species were identified to the lowest taxonomical level using local fishing guides^100^ or by consulting an expert if needed.

### Habitat structure and composition

Habitat composition was determined by analyzing reference images of substrates from each BRUVS. Reference images were overlapped with a 400 evenly-space red dot matrix that was used to estimate the proportion of each substrate available (i.e. number of dots that felt in each substrate type relative to the total number of overlapping points on identifiable substrates) (Fig. S2). Classification and estimation of each substrate cover percentage was made by two independent observers. The substrate cover, hereafter referred to habitat composition, was categorized in the following eight major habitats: bare rock with encrusting organisms, reef-building coral, bleached coral, rock/turf, macroalgae, sand/rubble (including small rubble), *Caulerpa sertularioides* (invasive algae), and others (i.e. anemones, barnacles, cyanobacteria, octocoral, sponges, softcoral and tunicates). A qualitative scale (1–3; low to high) was used to assess the degree of topographic complexity, visibility and depth of field (i.e. low values were assigned when footage was obstructed by a rock or other kind of substrate, or when camera was facing down/up) for each reference image (Fig. S3). BRUVS with very low visibility and depth of field (average of both parameters between two observers: < 1.5) were removed from the analyses. A protocol was created in order to standardize the substrate cover analysis methodology and decrease subjectivity among observers (see Table S6 for more details). Although results from this analysis may be restricted by the field of view and visibility of each BRUVS, they provided reliable data to evaluate species-habitat associations at the BRUVS level.

We used a principal component analysis (PCA), by constraining habitat scores, to display only the variation among BRUVS that could be explained by the percent cover of major habitat types^101^. This reduced the number of habitat components that explained > 70% of the variability amongst BRUVS into two major principal scores: (1) bare rock with incrusting organisms (PC1); (2) rock/turf to sand/rubble (PC2) (Table S7). The PCA was analyzed using the *RDA* function from the *vegan* library in R statistical package v.3.6.2^102^.

### Elasmobranch assemblages across sites

General patterns of elasmobranch species composition and relative abundance were examined using BRUVS across 11 sites of the Pacific waters of Costa Rica between December 2016 and June 2019 (Fig. 1). From our surveys, 30 BRUVS had low visibility and/or low depth of field, and therefore, were removed from the analysis. The remaining 430 BRUVS consisted of 362 benthic stations (sampling effort: 604.5 h) and 68 mid-water stations (sampling effort: 106.0 h) (Table S4). Sites with < 15 BRUVS from the North Pacific (Flamingo, Culebra Bay and Nicoya Peninsula) and South Pacific (Marino-Ballena and Golfo Dulce) regions were removed from the analyses.

At the BRUVS level, we determined the proportion of stations that recorded elasmobranchs at each site and region (e.g. Cocos Island, North and South Pacific). Both benthic and mid-water BRUVS detected a similar shark richness (11 and 10 species, respectively), whereas rays were more common on benthic (16 species) than mid-water BRUVS (6 species) (Table S4). Given that fewer mid-water BRUVS (N = 68) were deployed across sites compared to benthic BRUVS (N = 362; Table S3), elasmobranch assemblages from both benthic and mid-water BRUVS were pooled for analyses. Due to logistic reasons, there was some variability in deployment soak times (Table S3). However, most BRUVS had deployments that lasted > 80 min (Table S3). A recent study found that 77 min is an optimal soak time for BRUVS surveys of reef-associated elasmobranchs (95% of species sightings occur between 63 and 77 min), with longer deployments not having significant effects on species richness^85^. Based on these findings, we used the time to first sighting (TFS—time elapsed between the start of the sampling period and the first record of a particular species in the field of view) to assess any potential issues with different sampling effort (soak times) across sites. First, we calculated the proportion of TFS events for all species recorded at each site by time. Then, we used the empirical cumulative density function (ECDF, a step function) to estimate the fraction of observations of TFS that were less than or equal to a specified value. The ECDF plots were used to find the 75th and 95th percentiles of TFS for all species by site (Fig. S1).

Elasmobranch species captured by our BRUVS were classified as filter-feeders, small predators (species with maximum body sizes < 1.5 m) or large predators (species with maximum body sizes > 1.5 m) in order to examine changes in the trophic structure across sites. Since our study did not use stereo-BRUVS our ability to estimate individual size of elasmobranchs was limited. Therefore, our classification system was based on average size-estimates from underwater visual surveys conducted at the same study sites (Espinoza unpublished data), the maximum body size of the species^103^ and following the criteria regarding the functional groups classification stated by Refs.^104,105^. Therefore, any size estimates provided are restricted to the proportion of species detected at the site during underwater visual surveys and did not include any abundance information.

In order to reduce biases associated to bait dispersal among close BRUVS units or the wide-ranging movement patterns of some captured species, BRUVS that were deployed simultaneously at the same site and date were pooled together and treated as independent samples. Therefore, the maximum MaxN of each elasmobranch species per date was summed across sites. To standardize sampling effort across sites, the MaxN was divided by the soak time for each site/date, and expressed as catch per unit effort (CPUE; MaxN hrs^−1^). A non-metric multidimensional scaling (nMDS) ordination plot followed by a Permutational Multivariate Analysis of Variance (PERMANOVA; 1000 permutations) was used to examine differences in elasmobranch assemblages among sites. The function *ordiellipse* from the *vegan* library in R v.3.6.2^102^ was used to display the standard deviation of points from each site. A wrapper function (*pairwise.adonis*) that returns adjusted *p* values for multilevel pairwise comparisons from the *vegan* library in R v.3.6.2 was used to determine sites that were significantly different from each other. A Bray–Curtis dissimilarity matrix was constructed with the transformed elasmobranch species CPUE in columns (squared root MaxN hrs^−1^) and sites/dates in rows to reduce the influence of highly abundant species^106^. Using the same species-site matrix from nMDS analysis, we used the function *annHeatmap2* from the *Heatplus* library in R v.3.6.2^102^ to examine how the samples (i.e. BRUVS deployed at each site on different dates) clustered by site, and also the level of co-occurrence among species. For this analysis, we calculate the Bray–Curtis dissimilarity matrix on the full data set and used an average linkage hierarchical clustering for rows (samples) and columns (species). Ten species were removed from the analysis (*Diplobatis ommata*, *Narcine entemedor*, *Styracura pacifica*, *Urotrygon aspidura*, *Mobula Japonica*, *M. munkiana*, *M. tarapacana*, *Rhincodon typus*, *Pseudobatos glauca*, *P. planiceps*) because of their low frequency of occurrence (they were sighted in less than 5% of the stations). The resulting heat map allowed identifying dominant species, as well as clustering among rows and columns.

### Drivers of elasmobranch abundance and diversity

Poisson and negative binomial generalized linear models (GLMs) were used to assess how species richness and relative abundance (MaxN) were influenced by region (e.g. Isla del Coco, North and South Pacific), habitat protection (no-take vs. open fishing sites), elasmobranch group (sharks vs. rays), environmental drivers (temperature and depth) and habitat composition (e.g. PC1 and PC2). Based on our data, Poisson models outperformed negative binomial models. Only Isla del Coco, Islas Murciélago and Isla del Caño were considered true no-take MPAs, as some types of recreational and artisanal fishing are still allowed in Santa Elena Bay. Sampling effort (hr) was included as an offset to account for variability in soak time at each site. The performance of Poisson models were compared using maximum likelihood ratio tests and Akaike’s information criterion (AIC) of nested models. To determine the importance of the predictors from selected models, the difference in AIC with and without each term was computed using likelihood ratio tests. These analyses were done using the libraries *pscl*, *MuMIn* and *lmtest* from R v.3.6.2^102^.

An ordinal logistic regression model was used to investigate the effect of habitat protection on the number of BRUVS that recorded small or large predators using the *polr* from the MASS library in R v.3.6.2^102^. For this analysis, all elasmobranch species classified as small or large predators were summed across BRUVS from sampling sites belonging to the same category of habitat protection, and a presence-absence matrix was created for these two predatory groups (small and large predators). Filter-feeder species were excluded from the analyses. From the presence-absence matrix, we classified the predatory groups as − 1 (if only small predators were detected at each station), 0 (if there were both small and large predators detected at each station) or 1 (if only large predators were detected). Following the ordinal logistic analysis, we calculated the predicted probabilities of each event.

## Supplementary information

Supplementary Figure S1.

Supplementary Figure S2.

Supplementary Figure S3.

Supplementary Table S1.

Supplementary Table S2.

Supplementary Table S3.

Supplementary Table S4.

Supplementary Table S5.

Supplementary Table S6.

Supplementary Table S7.
