# Supplementary material for: Monitoring elasmobranch assemblages in a data-poor country from the Eastern Tropical Pacific using baited remote underwater video stations
Source: Sci Rep. 2020 Oct 14;10:17175. doi: 10.1038/s41598-020-74282-8 (PMC7560706; doi:10.1038/s41598-020-74282-8)
Supplement: Supplementary file 1 — Supplementary Figure S1. [file 41598_2020_74282_MOESM1_ESM.docx]

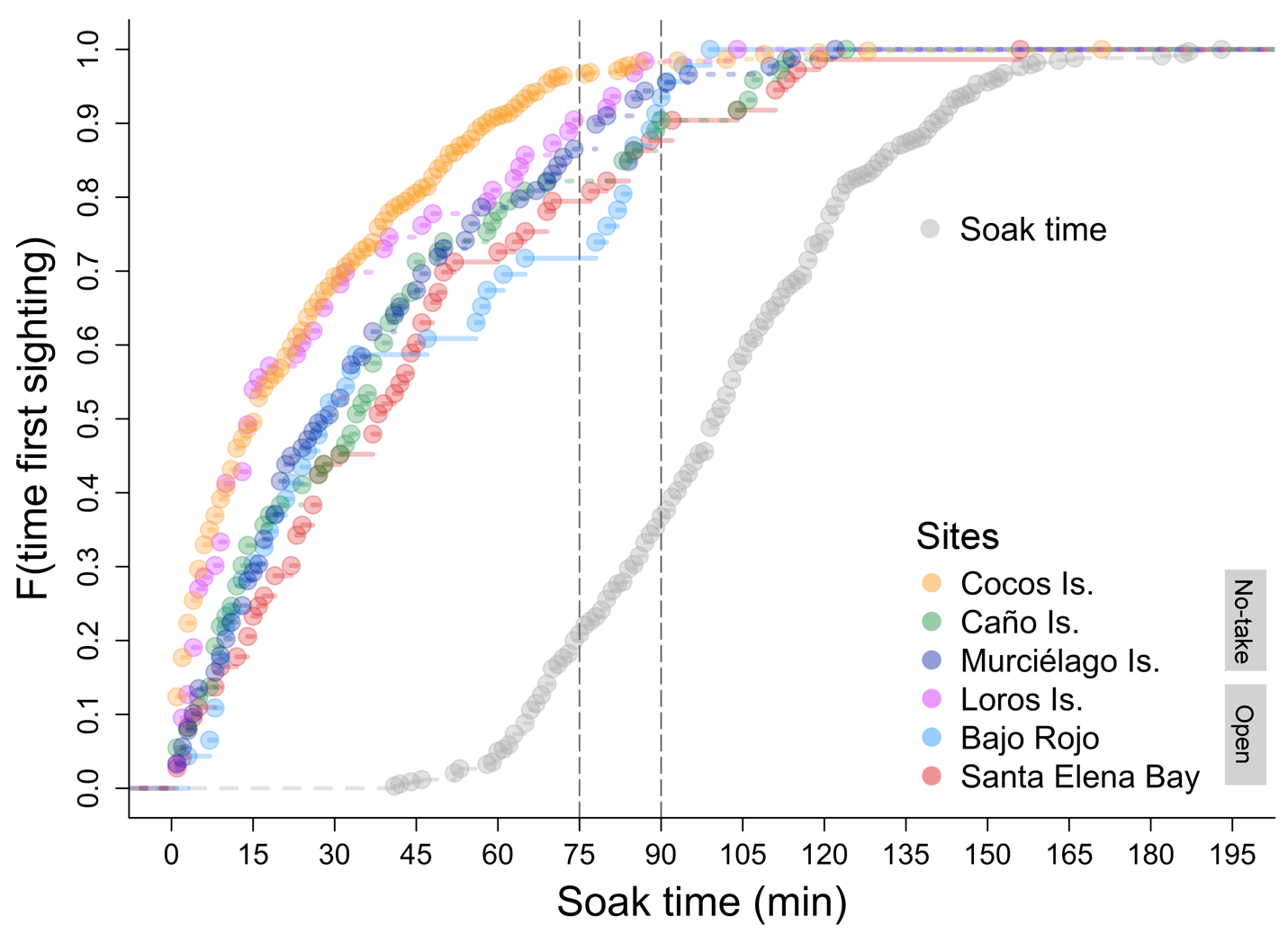


Figure S1. Empirical cumulative density functions (ECDF) of the time to first sighting (TFS) for all elasmobranch species by site. The ECDF for soak time (grey dotted line) is also shown. Vertical dotted lines on the x-axis represent a soak time of 75 and 90 min.
