# Supplementary material for: Monitoring elasmobranch assemblages in a data-poor country from the Eastern Tropical Pacific using baited remote underwater video stations
Source: Sci Rep. 2020 Oct 14;10:17175. doi: 10.1038/s41598-020-74282-8 (PMC7560706; doi:10.1038/s41598-020-74282-8)
Supplement: Supplementary file 2 — Supplementary Figure S2. [file 41598_2020_74282_MOESM2_ESM.docx]

**
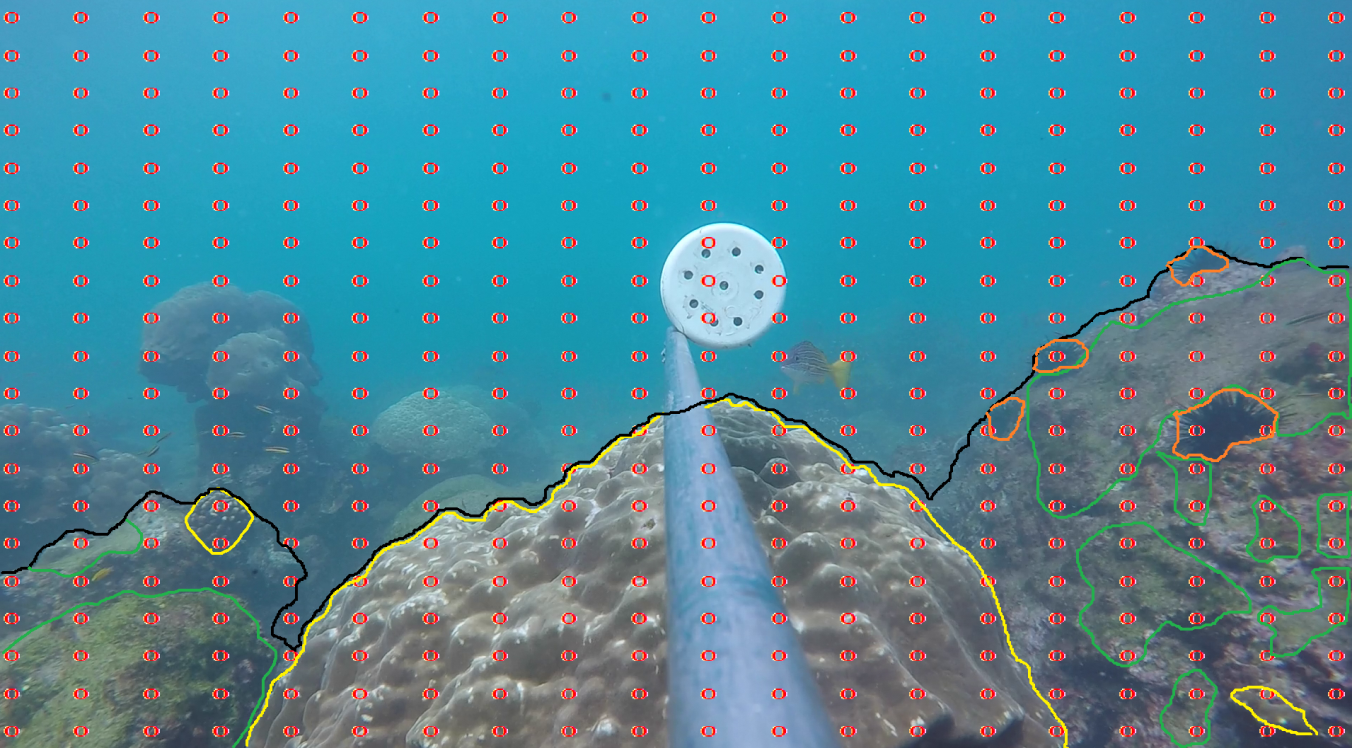
Figure S2.** Example of an image extracted from a Baited Remote Underwater Video Station at Cocos Island behind a matrix template of 400 dots. Colors indicate diferent cover categories selected in this study: coral reef (yellow), turf (green), sea urchin (orange), bare rock with encrusting organisms (the rest of the substrate without any color). The black line divides the matrix dots that fall over substrate categories included in the cover analysis (below the line) from the dots that are included in the “background” category and therefore will determine the field of view of the image. Cover percentage of each substrate category (step 3 at Table S7) would be the following: coral reef (n=73, 18.2%), turf (n=52, 13%), sea urchin (n=3, 0.75%), bare rock with encrusting organisms (n=31, 7.75%) and background (n=241, 60.2%). Qualitative scales following the standardized criteria of this study (Table S7; section 4) would be: visibility (3-high), topographic complexity (3-high), field of view (2-medium).
