# Supplementary material for: Monitoring elasmobranch assemblages in a data-poor country from the Eastern Tropical Pacific using baited remote underwater video stations
Source: Sci Rep. 2020 Oct 14;10:17175. doi: 10.1038/s41598-020-74282-8 (PMC7560706; doi:10.1038/s41598-020-74282-8)
Supplement: Supplementary file 3 — Supplementary Figure S3. [file 41598_2020_74282_MOESM3_ESM.docx]

**
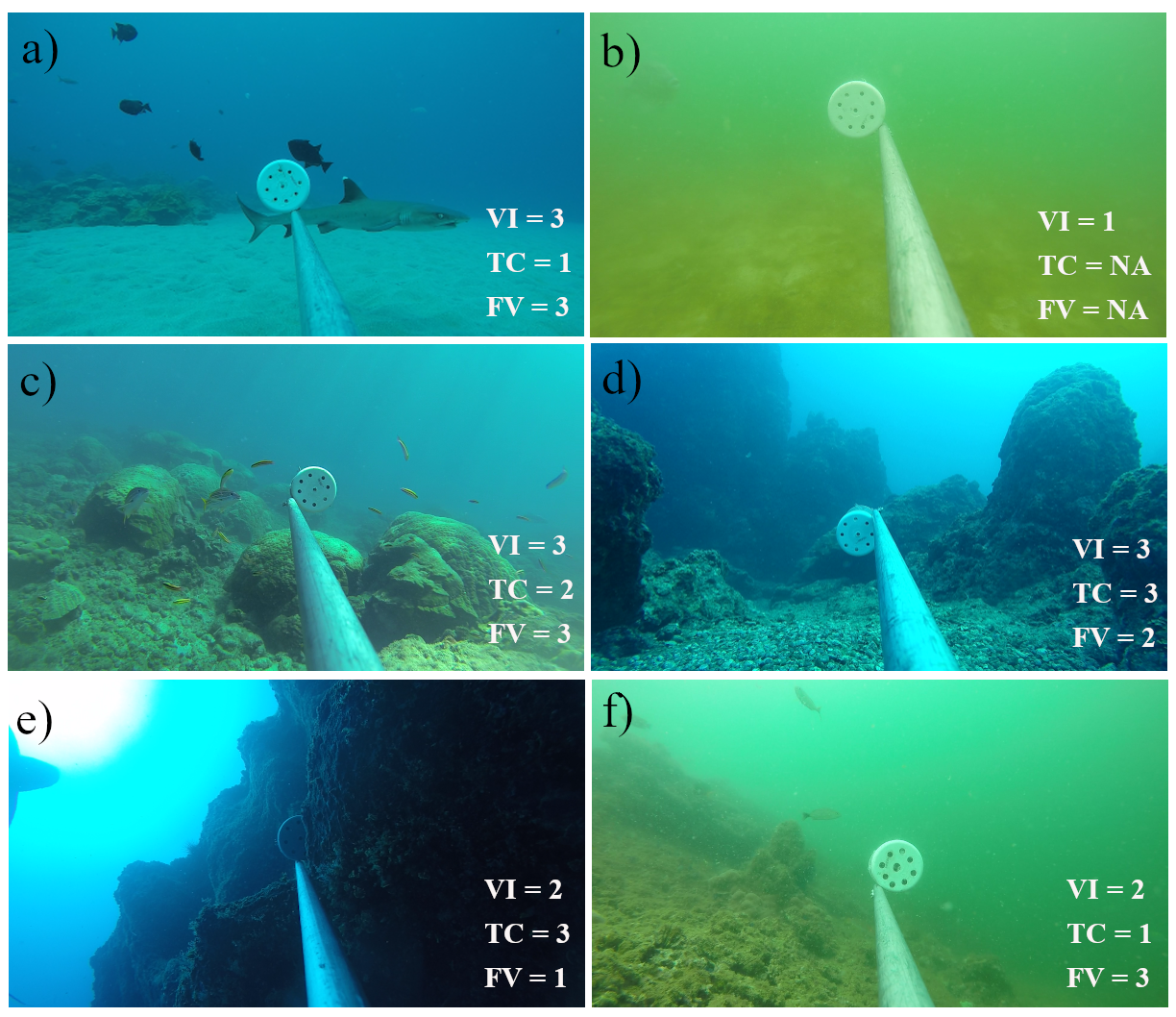
Figure S3.** Comparison of a qualitative scale analysis among reference images following the standardized criteria stablished in the substrate cover protocol created for this study. Visibility (VI), topographic complexity (TC) and field of view (FV) are analyzed at each image with a qualitative scale of 1 (low), 2 (medium) or 3 (high). The FV of each image is determined according to the number of points in the template that felt into the "Background" category (Table S7; section 3.3). When VI is to low, as shown in the image b), then TC and FV are not evaluated (“NA”). This image would be excluded from the study if both observers assign low values of VI and FV (Table S7; section 5).
