# Supplementary material for: Monitoring elasmobranch assemblages in a data-poor country from the Eastern Tropical Pacific using baited remote underwater video stations
Source: Sci Rep. 2020 Oct 14;10:17175. doi: 10.1038/s41598-020-74282-8 (PMC7560706; doi:10.1038/s41598-020-74282-8)
Supplement: Supplementary file 4 — Supplementary Table S1. [file 41598_2020_74282_MOESM4_ESM.docx]

Table S1. Generalized linear models ranked by AIC for species richness (top) and relative abundance (MaxN). Models were fitted with a Poisson distribution and included the following factors: region (Cocos Island, North and South Pacific), protection status (no-take and open to fishing), group (sharks and rays), depth, temperature and habitat component scores – PC1 and PC2). Soak time (hr) was used as an offset in the models. Best models are highlight in bold.

| Rank | Model | df | AIC |
| --- | --- | --- | --- |
| **1** | **richness ~ region + protection + group + depth + region × group + protection × group, offset = soak time** | **420** | **1883** |
| 2 | richness ~ region + protection + group + depth + PC1 + region × group + protection × group, offset = soak time | 419 | 1882 |
| 3 | richness ~ region + protection + group + depth + temperature + PC1 + region × group + protection × group, offset = soak time | 418 | 1880 |
| 4 | richness ~ region + protection + group + depth + temperature + PC1 + PC2 + region × group + protection × group, offset = soak time | 417 | 1882 |
| 5 | richness ~ 1, offset = soak time | 428 | 2133 |
| Rank | Model | df | AIC |
| **1** | **MaxN ~ region + protection + group + depth + PC2 + region × group + protection × group, offset = soak time** | **419** | **4523** |
| 2 | MaxN ~ region + protection + group + depth + region × group + protection × group, offset = soak time | 420 | 4527 |
| 3 | MaxN ~ region + protection + group + depth + temperature + PC2 + region × group + protection × group, offset = soak time | 418 | 4524 |
| 4 | MaxN ~ region + protection + group + depth + temperature + PC1 + PC2 + region × group + protection × group, offset = soak time | 417 | 4525 |
| 5 | MaxN ~ 1, offset = soak time | 428 | 6913 |
