# Supplementary material for: Monitoring elasmobranch assemblages in a data-poor country from the Eastern Tropical Pacific using baited remote underwater video stations
Source: Sci Rep. 2020 Oct 14;10:17175. doi: 10.1038/s41598-020-74282-8 (PMC7560706; doi:10.1038/s41598-020-74282-8)
Supplement: Supplementary file 5 — Supplementary Table S2. [file 41598_2020_74282_MOESM5_ESM.docx]

Table S2. Summary of elasmobranchs recorded in the Pacific of Costa Rica using different methods. Environment: P – pelagic; D – demersal; R – reef-associated; SG – sand and gravel; M – mud.

| Environment | Species | Trawling^35^ | Submarine^41^ | Longline^16^ | Diving^42^ | Diving^48^ | This study |
| --- | --- | --- | --- | --- | --- | --- | --- |
| P | *Alopias pelagicus* |  |  | X |  |  |  |
| P | *Alopias vulpinus* |  |  | X |  |  |  |
| P (R, S, M) | *Carcharhinus albimarginatus* |  |  |  | X |  | X |
| P (R) | *Carcharhinus falciformis* |  |  | X | X |  | X |
| P (R) | *Carcharhinus galapagensis* |  |  |  | X |  | X |
| P / D (R, SG, M) | *Carcharhinus leucas* |  |  | X |  |  | X |
| P / D (R, SG, M) | *Carcharhinus limbatus* |  |  | X | X |  | X |
| P | *Carcharhinus longimanus* |  |  | X |  |  |  |
| D (R, SG) | *Carcharhinus melanopterus* |  |  |  |  |  | X |
| P / D (R, SG, M) | *Galeocerdo cuvier* |  |  | X | X |  | X |
| P | *Nasolamia velox* |  |  | X |  |  |  |
| P | *Prionace glauca* |  |  | X |  |  |  |
| P / D (SG, M) | *Rhizoprionodon longurio* | X |  | X |  |  |  |
| D (R, SG) | *Triaenodon obesus* |  | X |  | X | X | X |
| D (R, SG) | *Hypanus dipterurus* |  | X | X |  | X |  |
| D (R, SG, M) | *Hypanus longus* | X |  | X |  | X | X |
| D (R, SG, M) | *Taeniurops meyeni* |  | X |  | X |  | X |
| D (R, SG) | *Echinorhinus cookei* | X | X |  |  |  |  |
| D (R, SG, M) | *Ginglymostoma unami* |  |  | X |  | X | X |
| D (R, SG) | *Gimnura marmorata* | X |  |  |  |  |  |
| P (R) | *Mobula birostris* |  | X |  | X |  | X |
| P (R) | *Mobula japanica* |  |  | X |  |  | X |
| P (R) | *Mobula munkiana* |  |  |  |  |  | X |
| P (R) | *Mobula tarapacana* |  | X | X | X |  | X |
| P (R) | *Mobula thurstoni* |  |  | X |  |  |  |
| P / D (R, SG, M) | *Aetobatus laticeps* | X | X | X | X |  | X |
| P / D (R, SG, M) | *Rhinoptera steindachneri* | X |  |  |  |  | X |
| P / D (R, SG, M) | *Diplobatis ommata* | X |  |  |  |  | X |
| D (SG, M) | *Narcine entemedor* | X |  |  |  |  | X |
| D (R, SG, M) | *Narcine vermiculatus* | X |  |  |  |  |  |
| D (SG, M) | *Odontaspis ferox* |  | X |  |  |  |  |
| D (SG, M) | *Styracura pacifica* |  |  |  |  |  | X |
| P | *Pseudocarcharias kaaharai* |  |  | X |  |  |  |
| D (SG, M) | *Rostroraja cortezensis* | X |  |  |  |  |  |
| D (SG, M) | *Rostroraja equatorialis* | X |  |  |  |  |  |
| D (SG, M) | *Rostroraja velezi* | X |  |  |  |  |  |
| P (R) | *Rhincodon typus* |  | X |  | X | X | X |
| D (SG, M) | *Pseudobatos glaucostigma* |  |  |  |  |  | X |
| D (SG, M) | *Pseudobatos leucorhynchus* | X |  |  |  |  |  |
| D (SG, M) | *Pseudobatos planiceps* |  |  |  |  |  | X |
| D (SG, M) | *Pseudobatos prahli* |  |  |  |  |  | X |
| D (SG, M) | *Sphyrna corona* |  |  | X |  |  |  |
| P / D (R, SG, M) | *Sphyrna lewini* | X | X | X | X |  | X |
| D (SG, M) | *Sphyrna tiburo* |  |  | X |  |  |  |
| P | *Sphyrna zygaena* |  |  | X |  |  |  |
| D (R, SG, M) | *Squatina californica* | X |  |  |  |  |  |
| D (SG, M) | *Tetronarce peruana* | X | X |  |  |  |  |
| D (R) | *Mustelus henlei* | X |  | X |  |  |  |
| D (SG, M) | *Mustelus lunulatus* | X |  | X |  |  |  |
| D (R, SG, M) | *Zapterix xyster* | X |  |  |  |  | X |
| D (R, SG, M) | *Urobatis halleri* |  |  |  |  |  | X |
| D (SG, M) | *Urotrygon aspidura* | X |  |  |  |  | X |
| D (SG, M) | *Urotrygon chilensis* | X |  |  |  |  | X |
| D (SG, M) | *Urotrygon cimar* | X |  |  |  |  |  |
| D (SG, M) | *Urotrygon munda* | X |  |  |  |  |  |
| D (SG, M) | *Urotrygon nana* | X |  |  |  |  |  |
| D (SG, M) | *Urotrygon rogersis* | X |  |  |  |  |  |
