# Supplementary material for: Monitoring elasmobranch assemblages in a data-poor country from the Eastern Tropical Pacific using baited remote underwater video stations
Source: Sci Rep. 2020 Oct 14;10:17175. doi: 10.1038/s41598-020-74282-8 (PMC7560706; doi:10.1038/s41598-020-74282-8)
Supplement: Supplementary file 6 — Supplementary Table S3. [file 41598_2020_74282_MOESM6_ESM.docx]

Table S3. Spatial and temporal drivers determining the survey effort (number of BRUVS per year) necessary to measure changes on reef associated elasmobranch species (e.g. richness, abundance and distribution) using Baited Remote Underwater Video Stations (BRUVS) in tropical marine ecosystems. Cocos Island National Park (Costa Rica) is presented as a case of study.

| **Drivers** | **Description** | **Recommendations** | **Case of study** |
| --- | --- | --- | --- |
| SITES | Number of sampling sites | The selection of which and how many sites should be made according to the research’s objective and the amount of resources and time available. | Cocos Island |
| HABITAT TYPES | Habitat types that need to be surveyed to answer the research question | Within each site, the number of habitats that are or could potentially be used by the species of interest should be identified. Although BRUVS should be distributed preferentially covering the habitats available, zoning of the study area can also be done according to the different management categories or other spatial drivers of research and/or management interest. | 1. Coastal coral reefs 2. Coastal rocky reefs 3. Pinnacles 4. Bays |
| HABITAT COVERAGE | Number of replicates for an adequate representation of each habitat and depth ranges | The number of independent replicates (either single or multiple BRUVS deployed at the same time) should be selected according to the extent of each habitat and its depth ranges. Ideally, BRUVS should be deployed randomly covering the full extent of each habitat and its depth levels | Coastal coral reefs = 12 BRUVS  Coastal rocky reefs = 12 BRUVS  Pinnacles = 12 BRUVS  Bays = 8 BRUVS  *Total BRUVS = 44* |
| SEASONALITY | Survey frequency along the year to account for seasonal variability or other periodic variations along the year | Sampling during the same time of the year (e.g. same month) over different years is the first premise that should be accomplished to make valid comparisons among years. However, if enough time and resources are available, sampling at different times of the year would be ideal to account for seasonal variability. The same number of replicates should be obtained at each season to obtain meaningful comparisons between seasons. | Dry season (Dec – Jun) = 44 BRUVS  Rainy season (Jul – Nov) = 44 BRUVS  *Annual BRUVS = 88 BRUVS* |
| PERIODICITY | Long-term survey periodicity | When possible, monitoring should include a temporal component to detect changes of the focal species over time. We recommend an annual survey periodicity for as long as possible, especially if population trends want to be evaluated. | Annual survey covering dry and rainy seasons for a 10-year period (more if possible). |
