# Supplementary material for: Monitoring elasmobranch assemblages in a data-poor country from the Eastern Tropical Pacific using baited remote underwater video stations
Source: Sci Rep. 2020 Oct 14;10:17175. doi: 10.1038/s41598-020-74282-8 (PMC7560706; doi:10.1038/s41598-020-74282-8)
Supplement: Supplementary file 7 — Supplementary Table S4. [file 41598_2020_74282_MOESM7_ESM.docx]

Table S4. Main variables and common standards to guide the use of Baited Remote Underwater Video Stations (BRUVS) when surveying coral / rocky-reef associated elasmobranch populations in tropical marine ecosystems.

| **Variables** | **Standards** |
| --- | --- |
| REPLICATES | The number of replicates should allow an adequate spatial/temporal coverage of surveyed habitats whilst meeting the study objectives (see Table S5) |
| BAIT | *Bait species:* locally available oily fish species such as sardines (Clupeidae) or mackerels (Scombridae).  *Bait consistency:* chopped/crushed pieces (5 cm) to maximize bait odor dispersion  *Bait quantity:* 1–1.5 kg of bait for deployments between 60–90 min.  *Bait container*: perforated PVC container (holes diameter 1–2 cm) to resist shark bites and avoid fishes feeding on the bait. |
| DISTANCE | Ideal distance between independent BRUVS replicates depends on species of interest, fish behavior, current speed and direction, influence of tides, time of day, and bait used ^84^. We recommend a distance of at least 300 m between BRUVS; ideally 500 m. |
| SOAK TIME | Optimal soak times for BRUVS on reef-associated elasmobranch species are typically 60 – 80 minutes ^85^. |
| DEPTH RANGE | It is important to cover all depth ranges of each habitat in order to obtain valuable data on elasmobranchs vertical distribution. Depth ranges in BRUVS studies using GoPro cameras typically go from 3 m to 40 m (maximum housing depth capacity). |
| TIME | 1 hr after sunrise and 2 hr before sunset if differential crepuscular behavior of species want to be avoided |
| BRUVS PLACEMENT | BRUVS placement at each survey site should be carefully analyzed in order to reduce risk of entanglement and maximize the field of view of resulting videos. Using mid-water BRUVS as presented in this study favors the fulfillment of both requirements. |
| FIELD DATA | Latitude / longitude, date, time and depth are essential field data to record for each BRUVS deployment. We recommend adding an identification number / letter to each station and each camera. Therefore, each replicate can be uniquely identified using this format: *ID station + ID camera_site_date (e.g. “S2C5_WaferBay_2019-10-01”).* |
| ADDITIONAL DATA | Environmental data that can potentially affect species surveyed should be measured if possible. We recommend prioritizing environmental drivers that may have an effect on a particular site (e.g. temperature, salinity, tide, dissolved oxygen, etc.). |
| CAMERA | *Type:* accessible, small and high definition cameras such as GoPro’s are recommended  *Orientation:* a horizontal camera arrangement to increase the field of view and facilitate species identification  *Settings:* 60 frames per second, 1080 p resolution and wide angle field of view |
