# Supplementary material for: Monitoring elasmobranch assemblages in a data-poor country from the Eastern Tropical Pacific using baited remote underwater video stations
Source: Sci Rep. 2020 Oct 14;10:17175. doi: 10.1038/s41598-020-74282-8 (PMC7560706; doi:10.1038/s41598-020-74282-8)
Supplement: Supplementary file 8 — Supplementary Table S5. [file 41598_2020_74282_MOESM8_ESM.docx]

Table S5. Summary of sampling effort by site. The number of benthic and mid-water baited remote underwater video stations (BRUVS) deployed and the number of shark and ray species detected per site are represented. Protection status: no-take marine protected areas and open to fishing sites.

| Protection  status | Main sampling  sites | N | Effort  (hr) | Soak time (min) | BRUVS | Benthic | Semi-pelagic |
| --- | --- | --- | --- | --- | --- | --- | --- |

|  |  |  |  | Range | Mean ± SD | Benthic | Semi-pelagic | Sharks | Rays | Sharks | Rays |
| --- | --- | --- | --- | --- | --- | --- | --- | --- | --- | --- | --- |

| No-take | Cocos Is. | 158 | 231.2 | 50.0 – 185.6 | 89.0 ± 28.0 | 121 | 37 | 8 | 4 | 8 | 2 |
| --- | --- | --- | --- | --- | --- | --- | --- | --- | --- | --- | --- |
| No-take | Murciélago Is. | 67 | 115.6 | 64.0 – 165.2 | 106.1 ± 23.8 | 58 | 9 | 6 | 9 | 2 | 0 |
| No-take | Caño Is. | 59 | 113.9 | 87.3 – 192.8 | 117.4 ± 19.8 | 54 | 5 | 3 | 3 | 3 | 1 |
| Open | Santa Elena Bay | 42 | 86.3 | 52.3 – 187.3 | 123.2 ± 34.1 | 35 | 7 | 1 | 8 | 0 | 3 |
| Open | Loros Is. | 38 | 61.6 | 57.7 – 142.7 | 99.8 ± 14.1 | 35 | 3 | 1 | 6 | 0 | 4 |
| Open | Bajo Rojo | 37 | 62.1 | 57.7 – 142.7 | 100.7 ± 21.6 | 32 | 5 | 2 | 5 | 0 | 2 |
| Protection  status | Additional  sites | N | Effort  (hr) | Soak time (min) | | BRUVS | | Benthic | | Semi-pelagic | |

|  |  |  |  | Range | Mean ± SD | Benthic | Semi-pelagic | Sharks | Rays | Sharks | Rays |
| --- | --- | --- | --- | --- | --- | --- | --- | --- | --- | --- | --- |

| Open | Flamingo | 15 | 23.7 | 68.0 – 152.3 | 94.7 ± 21.6 | 13 | 2 | 2 | 6 | 0 | 3 |
| --- | --- | --- | --- | --- | --- | --- | --- | --- | --- | --- | --- |
| Open | Golfo Dulce | 9 | 23.9 | 121.5 – 200.3 | 161.1 ± 32.2 | 9 | – | 0 | 2 | – | – |
| Open | Culebra Bay | 3 | 3.5 | 76.8 – 93.1 | 85.0 ± 11.5 | 3 | – | 0 | 2 | – | – |
| No-take | Marino-Ballena | 1 | 2 | 121.3 | – | 1 | – | 1 | 0 | – | – |
| Open | Nicoya Península | 1 | 1.5 | 90.0 | – | 1 | – | 0 | 0 | – | – |
