# Supplementary material for: Monitoring elasmobranch assemblages in a data-poor country from the Eastern Tropical Pacific using baited remote underwater video stations
Source: Sci Rep. 2020 Oct 14;10:17175. doi: 10.1038/s41598-020-74282-8 (PMC7560706; doi:10.1038/s41598-020-74282-8)
Supplement: Supplementary file 9 — Supplementary Table S6. [file 41598_2020_74282_MOESM9_ESM.docx]

**Table S6.** Summary of the protocol used in this study to analyze the substrate cover of each study site using reference images from Baited Remote Underwater Video Stations (BRUVS).

| **(1) IMAGE EXTRACTION** |
| --- |
| Extract an image (screenshot) that better reflects the substrate cover present at the monitoring site from each video file and save it as a PNG or JPEG file. Save the image under the same ID format used for that video (see Table S3 – Field data). |
| **(2) IMAGE PREPARATION** |
| Open the selected image with Paint (or another image editor program) and overlapp an evenly-space red dot matrix template that can be manually created with Word or another computer program. We used a matrix of 400 dots but another quantity of dots can be used as long as the dots completely cover the image and the same matrix is standardized for all images. The matrix image must have a transparent background so the image behind it can be visualized. This action can be done with Paint using the “Select” button and choosing the “transparent selection” option. The size of the matrix template should be manually adjusted until it covers the entire image to be analyzed. |
| **(3) IMAGE ANALYSIS – Cover percentage** |
| *3.1. Cover categories:* group the different types of substrate cover present in your study site into cathegories according to the objective of your study. In this study we used: bare rock with encrusting organisms, reef-building coral, bleached coral, turf, macroalgae, sand/rubble, invasive algaes, and others (i.e. anemones, barnacles, cyanobacteria, sponges, softcorals and tunicates)  *3.2. Cover percentage:* count the number of dots that fall in each cover category relative to the total number of dots on the matrix template (400 in this study). For example, 80 dots over coral reef / 400 = 0.2 or 20% of coral reef. See Figure S2 as an example.  *3.3. Background percentage:* count the number of dots that fall into the background of the image and consider them as another category. This category includes all those points on the matrix template that fall in the water column or on top of any type of substrate cover whose distance from the camera and / or visibility do not allow an appropriate identification of the cover category. The resulting percentage is important since it will determine the field of view of the image, which will be later used for image selection (next steps). Therefore, is important to define criteria that reduce the subjectivity among observers. In this study we used as a reference the difference between rock and turf. When these two categories could not be differentiated all dots over that substrate were considered “Background”. See Figure S2 as an example. |
| **(4) IMAGE ANALYSIS – Qualitative scales** |
| *4.1. Visibility:* define a qualitative scale from 1 to 3 (low to high) to asses the degree of visilibility of each image. Define an estimated range of visibility for each of the values of the scale. In this study we assigned 1 (low) when visibility was < 3m, 2 (medium) when 3 – 10m and 3 (high) when >10m. Reference images and additional criteria to better define each level of the scale (e.g. water color, camera orientation, distance at which objects can be distinguished, etc.) are important to reduce the subjectivity among observers. See Figure S3 as an example.  *4.2. Topographic complexity:* define a qualitative scale from 1 to 3 (low to high) to asses the degree rugosity or topographic complexity of each image. Define each value of the qualitative scale with many criterias as needed in order to reduce subjectivity among observers. In this study we assigned 1(low) when substrate was mainly flat or with few small rock formations, 2 (medium) when substrate was partially flat with some medium size rock or coral reef formations, 3(high) when substrate was mainly big rock formations or well-developed coral reef formations or a combination of both. See Figure S3 as an example.  *4.3. Field of view:* define a qualitative scale from 1 to 3 (low to high) to asses the field of view of each image according to the number of points (percentage) in the template that have fallen into the "Background" category. Lower backgroud percentage indicates that there was a rock formation or another object blocking the field of view of the video. In this study we assigned 1 (low), 2 (medium) and 3 (high) when image had < 33%, 33 -66 % and > 66% of background respectively. See Figure S3 as an example. |
| **(5) IMAGE SELECTION** |
| Calculate the average between visibility and field of view scale values from both observers. A unique final average value between 1 and 3 from these 4 parameters should be obtained. Set a limit value below which the image is not considered valid for analysis. In this study we considered images with a final average value < 1.5 not valid for analisis. |
