# Supplementary material for: Monitoring elasmobranch assemblages in a data-poor country from the Eastern Tropical Pacific using baited remote underwater video stations
Source: Sci Rep. 2020 Oct 14;10:17175. doi: 10.1038/s41598-020-74282-8 (PMC7560706; doi:10.1038/s41598-020-74282-8)
Supplement: Supplementary file 10 — Supplementary Table S7. [file 41598_2020_74282_MOESM10_ESM.docx]

Table S7. Summary of the results from the principal component analysis (PCA) of the eight major habitat types.

|  | PC1 | PC2 | PC3 |
| --- | --- | --- | --- |
| Eigenvalues | 0.040 | 0.029 | 0.013 |
| Proportion explained | 0.415 | 0.299 | 0.131 |
| Cumulative proportion | 0.415 | 0.714 | 0.845 |
| Habitat scores |  |  |  |
| Rock with incrusting organisms | 1.321 | 0.439 | 0.357 |
| Sand and rubble | -0.833 | 0.726 | 0.542 |
| Live coral | 0.028 | -0.025 | -0.144 |
| Death coral | 0.005 | -0.025 | -0.040 |
| Rock/turf | -0.026 | -1.022 | 0.547 |
| Macroalgae | -0.046 | 0.003 | -0.061 |
| *Caulerpa* *sertularoides*. | 0.031 | -0.008 | -0.144 |
| Others | 0.002 | -0.034 | -0.065 |
